# A genome-wide screen of Epstein-Barr virus proteins that modulate host SUMOylation identifies a SUMO E3 ligase conserved in herpesviruses

**DOI:** 10.1371/journal.ppat.1007176

**Published:** 2018-07-06

**Authors:** Carlos F. De La Cruz-Herrera, Kathy Shire, Umama Z. Siddiqi, Lori Frappier

**Affiliations:** Department of Molecular Genetics, University of Toronto, Toronto, Canada; University of Wisconsin-Madison School of Medicine and Public Health, UNITED STATES

## Abstract

Many cellular processes pertinent for viral infection are regulated by the addition of small ubiquitin-like modifiers (SUMO) to key regulatory proteins, making SUMOylation an important mechanism by which viruses can commandeer cellular pathways. Epstein-Barr virus (EBV) is a master at manipulating of cellular processes, which enables life-long infection but can also lead to the induction of a variety of EBV-associated cancers. To identify new mechanisms by which EBV proteins alter cells, we screened a library of 51 EBV proteins for global effects on cellular SUMO1 and SUMO2 modifications (SUMOylation), identifying several proteins not previously known to manipulate this pathway. One EBV protein (BRLF1) globally induced the loss of SUMOylated proteins, in a proteasome-dependent manner, as well as the loss of promeylocytic leukemia nuclear bodies. However, unlike its homologue (Rta) in Kaposi’s sarcoma associated herpesvirus, it did not appear to have ubiquitin ligase activity. In addition we identified the EBV SM protein as globally upregulating SUMOylation and showed that this activity was conserved in its homologues in herpes simplex virus 1 (HSV1 UL54/ICP27) and cytomegalovirus (CMV UL69). All three viral homologues were shown to bind SUMO and Ubc9 and to have E3 SUMO ligase activity in a purified system. These are the first SUMO E3 ligases discovered for EBV, HSV1 and CMV. Interestingly the homologues had different specificities for SUMO1 and SUMO2, with SM and UL69 preferentially binding SUMO1 and inducing SUMO1 modifications, and UL54 preferentially binding SUMO2 and inducing SUMO2 modifications. The results provide new insights into the function of this family of conserved herpesvirus proteins, and the conservation of this SUMO E3 ligase activity across diverse herpesviruses suggests the importance of this activity for herpesvirus infections.

## Author summary

The functions of many cellular proteins important for anti-viral responses and oncogenesis are controlled by modifications by small ubiquitin-like modifiers (SUMOs). Here we present the first screen of Epstein-Barr virus (EBV) proteins for those that can globally alter SUMO modifications of cellular proteins. We identify four distinct EBV proteins that increase SUMO modifications and one that decreases them. One of the SUMO upregulating proteins (SM) is conserved in other herpesviruses and we show that this activity is conserved in homologues from herpes simplex virus 1 (HSV1) and cytomegalovirus (CMV). We also show that these three homologues have SUMO E3 ligase activity in *in vitro* assays and that they bind SUMO and Ubc9, consistent with the expectation of SUMO E3 ligases. The results provide new insights into the functions and mechanisms of action of this family of herpesvirus proteins. Our study identifies the first SUMO E3 ligases for EBV, HSV1 and CMV and provides a new mechanism by which EBV can manipulate cellular processes, through global effects on cellular SUMOylation.

## Introduction

The functions of many cellular and viral proteins are controlled by the addition of small ubiquitin-like modifiers (SUMO), in the form of SUMO1 or SUMO2 or SUMO3 chains (referred to as SUMOylation). These modifications can affect protein stability or localization and can promote protein-protein interactions via binding of SUMO to SUMO-interacting sequences (SIMs) [1, 2]. SUMOylation controls many nuclear processes, including genome stability, gene expression, cell cycle progression, senescence and stress and innate immune responses [1, 3–7]. Not surprisingly based on these roles, SUMO signal transduction has been identified as a key factor in the development of several types of cancer; SUMOylation is highly upregulated in many cancers and some cancers have been shown to be dependent on a functioning SUMO system [1, 4]

DNA viruses manipulate many of the cellular processes regulated by SUMOylation and therefore SUMO pathways provide a mechanism to alter these processes. Several different mechanisms have been described by which viral proteins usurp the SUMO system. Cellular SUMOylation involves the SAE1/SAE2 E1 SUMO-activating enzyme, the Ubc9 E2 SUMO-conjugating enzyme and several different SUMO E3 ligases that mediate the interaction of charged Ubc9 with the target protein, facilitating the SUMO transfer [2]. Some viral proteins globally alter SUMOylation by hijacking Ubc9 [8]. For example, the E6 protein of human papillomavirus (HPV) type 16/18 lowers Ubc9 levels thereby globally decreasing SUMOylation [9]. Some viruses upregulate SUMOylation by encoding E3 SUMO ligases that function in conjunction with Ubc9. Two adenovirus proteins have been reported to have SUMO ligase activity; adenovirus E1B-55K induces SUMO1 modification of p53 [10, 11], while adenovirus E4-ORF3 induces SUMO3 modification of the transcription intermediary factor 1γ (TIF-1γ) [12]. In addition, the K-bZIP protein of Kaposi’s sarcoma-associated herpesvirus (KSHV) was found to be a SUMO2/3-specific E3 ligase that modifies itself as well as p53 and Rb [13].

Viruses can also downregulate SUMOylation by encoding SUMO-specific proteases (SENPs) or SUMO-targeted ubiquitin ligases (STUbLs); the latter which ubiquitylates SUMO-modified proteins leading to their degradation. Both human adenoviruses and vaccinia virus encode a SENP [14]. In addition, two distinct viral STUbLs have been identified; the ICP0 protein of herpes simplex virus 1 (HSV-1) and its homologue (ORF61) in varicella zoster virus, and the unrelated Rta protein of KSHV and its homologue in murine gammaherpesvirus 68 [15–19]. Like the cellular STUbL RNF4 [20], a major target of degradation of the viral STUbLs are promyelocytic leukemia (PML) and associated proteins since they are highly SUMO-modified [15, 16, 21]. Since PML nuclear bodies are part of the innate immune response that senses herpesvirus genomes and suppresses their lytic infection, these STUbLs have important roles in promoting lytic infection [16, 22, 23].

EBV is a wide-spread herpes virus that is a causative agent of several types of cancer, including Burkitt’s lymphoma, nasopharyngeal carcinoma and 10% of gastric carcinoma [24–26]. Life-long infection occurs due to the ability of EBV to alternate between latent modes of infection, with restricted viral gene expression, and lytic infection involving expression of an additional ~70 viral proteins. Together, these lytic proteins manipulate many cellular processes including innate immune responses, cell cycle progression and DNA damage responses, in order to promote cell survival and virion production [27, 28]. However, the functions of many EBV lytic proteins are unknown and even proteins with an assigned function may have additional unidentified roles. Given the importance of SUMO pathways in oncogenesis and cellular processes manipulated by EBV, it seems likely that some EBV proteins may function by targeting SUMO pathways.

Little is currently known about the interplay between EBV and SUMO systems. To date only one EBV protein, the latent membrane protein 1 (LMP1), has been shown to act directly on SUMOylation. LMP1 binds to Ubc9 and upregulates its activity, resulting in increased SUMOylation in EBV latent infection [29, 30]. This includes SUMOylation of KRAB-associated protein 1 (KAP1), which promotes its repression of the lytic immediate early promoters and lytic replication origin, thereby promoting latency [31]. In lytic infection, SUMO2/3 conjugates have been found to accumulate late in infection, which in part may be due to expression of a viral miRNA that downregulates RNF4 [32]. Presently no SUMO ligases, SENPs or STUbLs have been identified for EBV.

To investigate how EBV proteins impact cellular pathways, we previously generated an expression library for most of the EBV lytic proteins, and used the C-terminal FLAG tag on each protein to determine their subcellular localization [33]. These EBV proteins were then screened for the ability to disrupt or alter PML nuclear bodies [33], contribute to cell cycle arrest at the G1/S interface [28] and inhibit the cellular DNA damage response [34]; characteristics typical of EBV lytic infection. These screens have led to new functions for several EBV proteins demonstrating the utility of the approach. Here we screened the library of EBV proteins for those that globally affect cellular SUMO1 and SUMO2 modifications and identified a few EBV proteins that upregulate SUMOylation and one that downregulates SUMOylation. The downregulator is the Rta homologue of the KSHV and gammaherpesvirus 68 (γHV68) STUbLs, suggesting a conserved role for these protein in decreasing SUMOylation. We show that one of the SUMO upregulators (SM) has characteristics of a SUMO E3 ligase and that this activity is conserved in SM homologues in HSV1 (UL54/ICP27) and human cytomegalovirus (CMV; UL69), identifying the first SUMO E3 ligase for any of these herpesviruses.

## Results

### Identification of EBV proteins that globally affect cellular SUMOylation

**Fig 1:**
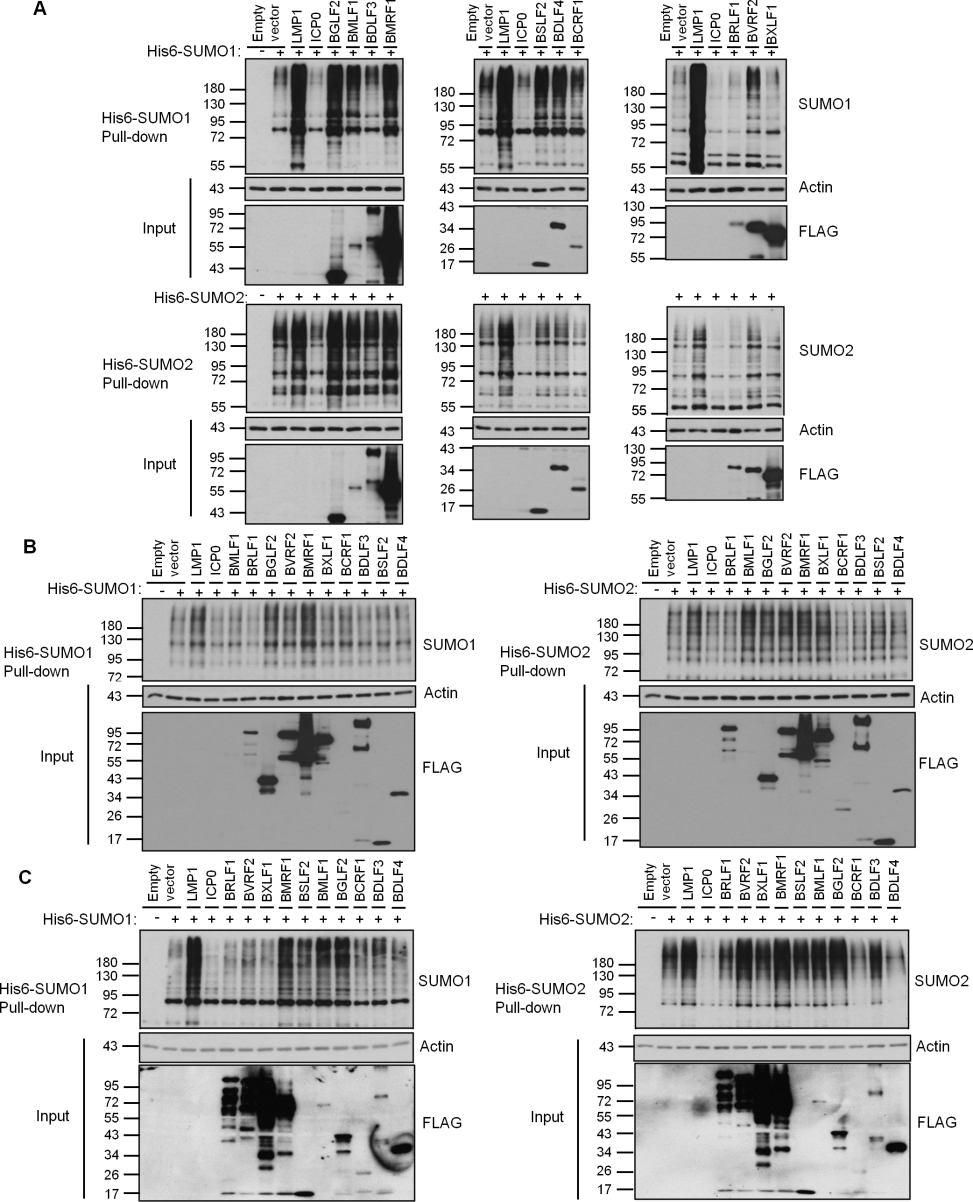
Screens of EBV proteins for global effects on cellular SUMO1 and SUMO2 modifications. A. 293T cells were co-transfected with plasmids expressing His6-SUMO1 (top panels) or His6-SUMO2 (bottom panels) and the indicated viral protein or empty vector control. B. HeLa cells stably expressing His6-SUMO1 (left panels) or His6-SUMO2 (right panels) were transfected with plasmids expressing the indicated viral protein or empty vector control. C. CNE2Z cells were co-transfected with plasmids expressing His6-SUMO1 (left panels) or His6-SUMO2 (right panels) and the indicated viral protein or empty vector control. In all cases, His6-tagged proteins were recovered from cell lysates on metal chelating resin under denaturing conditions (Pull-down panels) and immunoblotted for SUMO1 or SUMO2 as indicated. Samples of the lysates (Inputs) were also immunoblotted for actin and FLAG, to detect the FLAG-tagged EBV library proteins. Note that LMP1 and ICP0 did not contain FLAG-tags and hence are not seen in the FLAG blots.

**Fig 2:**
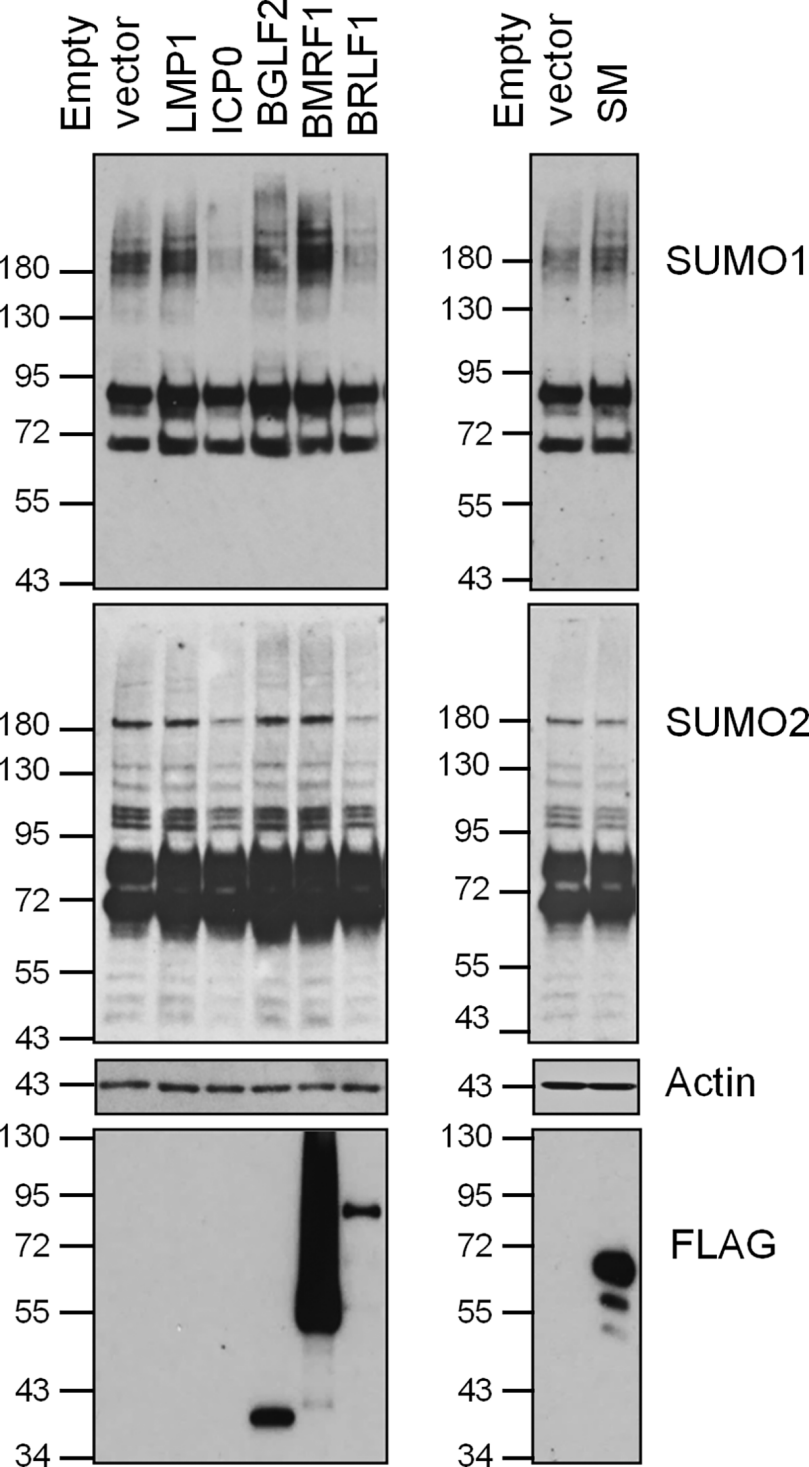
Effect of selected EBV proteins on endogenous SUMO1 and SUMO2 modifications. 293T cells were transfected with plasmids expressing the indicated viral protein or empty vector control. 36 hours later, cell lysates were generated and equal amounts were immunoblotted for SUMO1, SUMO2, FLAG and actin.

To identify EBV proteins that hijack cellular SUMOylation, we screened a library of 51 EBV lytic proteins for the ability to globally affect SUMO1 and SUMO2 modifications upon overexpression. Such a screen can identify proteins with intrinsic SUMOylation or SUMO degradation activities or that have prominent interactions with SUMO pathway proteins [15, 35–37]. Initial screens were performed in both 293T cells transiently expressing His6-tagged SUMO1 or SUMO2 and in HeLa cells containing integrated copies of His6-SUMO1 or His6-SUMO2 that express these proteins at close to endogenous levels [38]. In both cases, FLAG-tagged EBV proteins were expressed by transient transfection and, 36 hrs later, His-tagged proteins were recovered from cell lysates on metal chelating resin under denaturing conditions. Western blots were then performed using SUMO1 or SUMO2 antibodies to detect all proteins covalently modified by His-SUMO. The SUMOylation profile in the presence of each viral protein was compared to that with empty FLAG-tagged plasmid. In addition, we used the EBV LMP1 protein as a positive control for global upregulation of SUMOylation and the HSV1 ICP0 protein as a positive control for global downregulation of SUMOylated proteins. Examples of these controls and selected EBV proteins are shown in Fig 1 (see S1 Fig for confirmation of ICP0 and LMP1 expression). The up or down regulation of global SUMOylation was assessed by comparing the intensity of the ladder of SUMOylated proteins in the presence of the EBV protein to that of the empty plasmid, and results for all 51 EBV proteins in the two cell systems are summarized in Table 1 (with the degree of the effect indicated by the number of + signs for upregulation or– signs for downregulation). For proteins that appeared to affect global SUMOylation in 293T and HeLa cells, assays with transient expression of His6-SUMO1 or His6-SUMO2 were repeated in a nasopharyngeal carcinoma cell line (CNE2Z) relevant for EBV infection (Fig 1C and Table 1). We then looked for EBV proteins that consistently up or down regulated SUMO1 or SUMO2 modifications in all three cell systems. Six EBV proteins met this criteria and had moderate to high effects in at least one cell system (marked in grey in Table 1). Five of these increased SUMOylation while one (BRLF1 or Rta) decreased SUMOylation. Quantification of these effects is shown in Table 2. Ability to modulate SUMOylation did not correspond to the expression levels of the viral proteins, as BMLF1 and BRLF1 were expressed at relatively low levels and some highly expressed proteins (such as BXLF1) did not affect SUMOylation. In addition, increases in SUMOylation was not simply due to SUMOylation of the viral protein itself, as even when SUMOylation of the viral protein could be detected, it resulted in only a few discreet bands and not the high molecular weight smears seen in the SUMOylation screen (see examples in S2 Fig). Upregulation of SUMOylation by BGLF2, BMRF1 and SM and down regulation of SUMOylation by Rta was also confirmed by detecting endogenous SUMO levels in 293T cells (Fig 2).

**Table 1:** Screen of EBV Lytic proteins for global effects on cellular SUMOylation*.

Protein	Function	293T		HeLa		CNE2Z	
		S1	S2	S1	S2	S1	S2
BALF1	bcl-2 homolog	n/e	+	n/e	+	n/e	+
BALF2	ssDNA binding protein	n/e	n/e	+	n/e		
BALF3	Endonuclease; DNA packaging	n/e	n/e	n/e	n/e		
BARF1	secreted cell stimulatory factor	n/e	n/e	n/e	n/e		
BaRF1	ribonucleotide reductase small subunit	n/e	n/e	n/e	n/e		
BBLF1	Myristylated membrane protein	+	n/e	n/e	n/e		
BBLF2	part of BBLF2/3 protein	+	+	n/e	n/e	n/e	+
BBLF4	DNA helicase	n/e	n/e	+	n/e		
BBRF1	portal protein	n/e	+	n/e	+	n/e	n/e
BBRF2	uncharacterized HSV1 UL7 homologue	n/e	n/e	n/e	n/e		
BcRF1	TATT binding protein; late gene activator	n/e	n/e	n/e	n/e		
BCRF1	viral Interleukin 10	n/e	**-**	n/e	**-**	n/e	**-**
BDLF1	minor capsid protein	n/e	n/e	n/e	n/e		
BDLF2	glycosylated envelope protein	n/e	n/e	n/e	n/e		
BDLF3	envelope glycoprotein gp150	n/e	+	n/e	n/e	+	n/e
BDLF4	late gene expression	+	n/e	n/e	n/e	+	--
BDRF1	scaffold protein	n/e	+	n/e	+	n/e	n/e
BFLF2	DNA packaging	n/e	n/e	+	n/e		
BFRF1	viral egress	n/e	n/e	n/e	n/e		
BFRF2	uncharacterized HSV1 UL49 homologue	n/e	n/e	n/e	n/e		
BFRF3	small capsid protein	n/e	+	n/e	n/e		
BGLF1	DNA packaging; HSV1 UL17 homologue	n/e	n/e	n/e	n/e		
BGLF2	cell cycle & AP-1 modulator	+++	++	++	++	++	++
BGLF3	serine threonine kinase	n/e	+	n/e	+	n/e	n/e
BGLF4	uncharacterized HSV1 UL15 homologue	n/e	n/e	n/e	+		
BGRF1	uracil DNA glycosylase	+	n/e	n/e	n/e	+	n/e
BKRF3	scaffold protein	n/e	n/e	n/e	n/e		
BKRF4	Histone binding protein	+	n/e	n/e	+		
BLLF2	uncharacterized late gene	n/e	n/e	n/e	n/e		
BLLF3	dUTPase	+	n/e	n/e	n/e		
BLRF1	envelope glycoprotein, gN homologue	n/e	n/e	n/e	n/e		
BLRF2	uncharacterized tegument	n/e	n/e	n/e	n/e		
BMLF1	C-terminal part of SM/EB2	+++	+	n/e	++	++	++
BMRF1	DNA polymerase processivity factor	+++	++	++	++	++	++
BNLF2a	immune evasion; TAP inhibitor	n/e	n/e	n/e	+	n/e	n/e
BNLF2b	uncharacterized	n/e	n/e	n/e	n/e		
BORF1	minor capsid protein	n/e	n/e	n/e	n/e		
BORF2	Ribonucleotide-reductase, large subunit	n/e	+	n/e	n/e		
BRLF1	Rta transcriptional activator	**- -**	**-**	**-**	**-**	**-**	**-**
BRRF1	Na transcriptional activator	+	n/e	**-**	n/e		
BRRF2	viral egress	+	n/e	n/e	n/e	+	n/e
BSLF1	DNA Primase	n/e	n/e	n/e	n/e		
BSLF2	N-terminal part of SM/EB2	+	n/e	n/e	+	++	+
BSRF1	uncharacterized tegument protein	n/e	n/e	n/e	n/e		
BTRF1	uncharacterized	n/e	n/e	n/e	n/e		
BVLF1.5	uncharacterized	n/e	n/e	n/e	n/e		
BVRF1	uncharacterized tegument protein	+	n/e	n/e	n/e	n/e	+
BVRF2	autocatalytic scaffold protease	++	++	+	++	n/e	++
BXLF1	Thymidine kinase	n/e	n/e	n/e	++	n/e	+
BZLF1	Zta (ZEBRA) transcriptional activator	n/e	n/e	n/e	n/e		
SM (EB2)	mRNA binding and export	+++	n/e	+	n/e	+++	n/e

* EBV proteins that induce (+) or decrease (-) SUMO1 (S1) and SUMO2 (S2) modifications are indicated for three cell lines. The number of + or - indicates the degree of the effect. n/e = no effect. Proteins that gave consistent effects in all three cell lines with at least one moderate (++ or --) or higher effect are marked in grey.

**Table 2:** Fold change in global SUMOylation relative to empty vector control*.

	293T		HeLa		CNE2Z	
Protein	SUMO1	SUMO2	SUMO1	SUMO2	SUMO1	SUMO2
LMP1	4.63 ± 0.19	1.70 ± 0.16	2.01 ± 0.44	1.73 ± 0.05	2.25 ± 0.89	2.08 ± 0.95
ICP0	0.29 ± 0.12	0.26 ± 0.20	0.75 ± 0.05	0.74 ± 0.04	0.56 ± 0.07	0.17 ± 0.04
BGLF2	3.84 ± 0.69	2.0 ± 0.39	1.93 ± 0.73	1.65 ± 0.01	1.61 ± 0.28	1.91 ± 0.41
BMLF1	2.75 ± 0.46	1.26 ± 0.03	1.11 ± 0.09	1.56 ± 0.29	1.48 ± 0.07	1.76 ± 0.45
BMRF1	3.02 ± 0.03	1.74 ± 0.40	2.57 ± 0.12	1.65 ± 0.05	1.83 ± 0.57	2.07 ± 0.74
BRLF1	0.45 ± 0.47	0.54 ± 0.07	0.61 ± 0.03	0.65 ± 0.04	0.59 ± 0.08	0.73 ± 0.29
BVRF2	1.98 ± 0.08	1.98 ± 0.24	1.33 ± 0.21	1.58 ± 0.30	1.05 ± 0.06	1.71 ± 0.29
SM	4.52 ± 1.56	1.07 ± 0.45	1.80 ± 0.46	1.15 ± 0.10	2.62 ± 0.46	1.02 ± 0.29
UL54	1.15 ± 0.46	1.40 ± 0.19	1.02 ± 0.06	1.96 ± 0.17	1.24 ± 0.22	2.27 ± 0.13
UL69	2.48 ± 0.21	1.06 ± 0.46	1.50 ± 0.16	1.26 ± 0.25	2.0 ± 0.17	0.99 ± 0.37

*Average values from multiple experiments are shown +/- standard deviation.

### SUMO-associated properties of BRLF1

**Fig 3:**
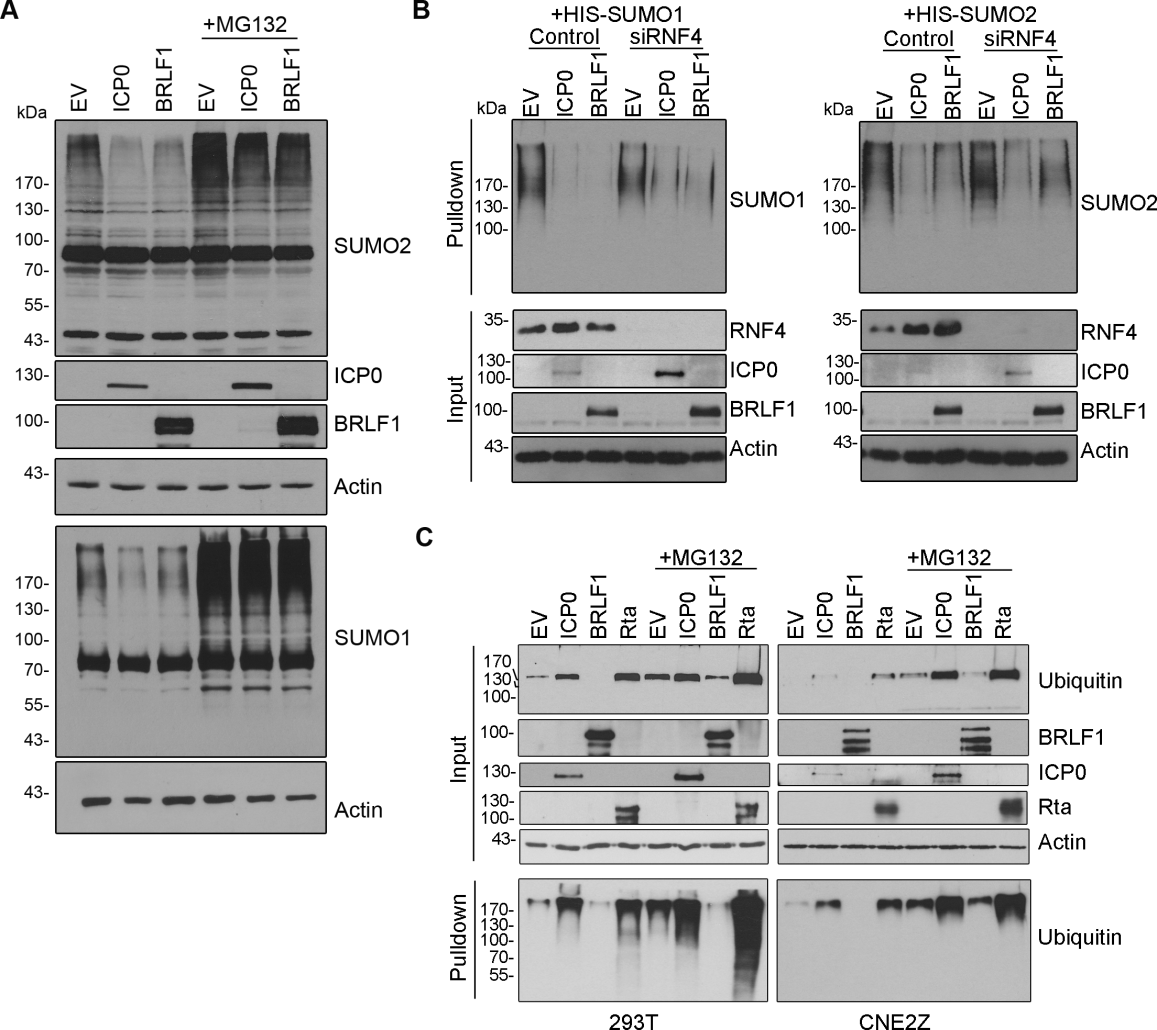
BRLF1 induces proteasomal-dependent loss of SUMOylated proteins without inducing ubiquitylation. A.293T cells were transfected with plasmids expressing ICP0, FLAG-BRLF1 or empty vector (EV) control, then treated with MG132 (+) or left untreated (-). 34 hours post-transfection, cell lysates were analysed by Western blotting using the antibodies against SUMO1, SUMO2, ICP0, FLAG (BRLF1) or actin. Samples for SUMO1 and SUMO2 blots were run on separate gels and the actin loading control is shown for each. B. Experiments in A (without MG132) were repeated in CNE2Z cells with (siRNF4) and without (Control) silencing of RNF4. C. 293T (left) or CNE2Z (right) cells were transfected with plasmids expressing either ICP0, FLAG-BRLF1, Strep-Rta or empty vector (EV) along with a plasmid expressing myc-his-ubiqutin. Cell lysates were Western blotted directly (input) with antibodies against myc (to detect ubiquitin conjugates), FLAG (for BRLF1), Strep (for Rta) or ICP0. Myc-his-ubiquitin containing proteins were isolated from the lysates on metal chelating resin (Pulldown) prior to blotting for myc.

BRLF1 was the only EBV protein that we found to consistently decrease the level of SUMOylated proteins with a moderate to high effect. This decrease was seen for both SUMO1- and SUMO2-modified proteins. This effect was further verified by expressing BRLF1 in 293T cells and blotting for endogenous SUMO (Fig 2). The observed decrease in SUMO1 and SUMO2 modified proteins was not due to effects on SUMO or Ubc9 transcripts, as the level of SUMO1, SUMO2 or Ubc9 mRNA was not significantly changed by Rta expression (S3 Fig). Since the KSHV homologue of BRLF1 (Rta) was shown to be a STUbL, we asked whether BRLF1 had characteristics consistent with a STUbL. Since STUbLs target SUMOylated proteins for proteasomal degradation, we asked whether the loss of SUMOylated proteins induced by BRLF1 could be restored by blocking the proteasome with MG132. As shown in Fig 3A, MG132 at least partly countered the loss of both SUMO1 and SUMO2-modified proteins that is induced by BRLF1, mirroring the results with the HSV1 STUbL, ICP0. Since BRLF1 has been reported to associate with the cellular STUbL, RNF4 [39], we also asked whether the loss of SUMOylated proteins caused by BRLF1 required RNF4. However, much like ICP0, BRLF1 was found to induce the loss of both SUMO1 and SUMO2-modified proteins in CNE2Z cells (relative to empty vector control) regardless of whether or not RNF4 was silenced (Fig 3B).

Rta proteins from KSHV and γHV68 have ubiquitin ligase activity that can be observed by upregulation of global cellular ubiquitylation upon their overexpression [15, 18, 40]. To determine if this was also true for BRLF1, we expressed it along with His6-myc-ubiquitin in both 293T and CNE2Z cells, with and without MG132 treatment, then recovered ubiquitylated proteins on metal chelating resin and detected them by Western blotting with anti-myc antibody (Fig 3C). While KSHV Rta and HSV1 ICP0 induced ubiquitylation in both cell lines both with and (to a lesser degree) without MG132, no induction of ubiquitylation was detected with BRLF1. This suggests that, unlike Rta and ICP0, BRLF1 induces the loss of SUMOylated proteins without endogenous ubiquitin ligase activity.

**Fig 4:**
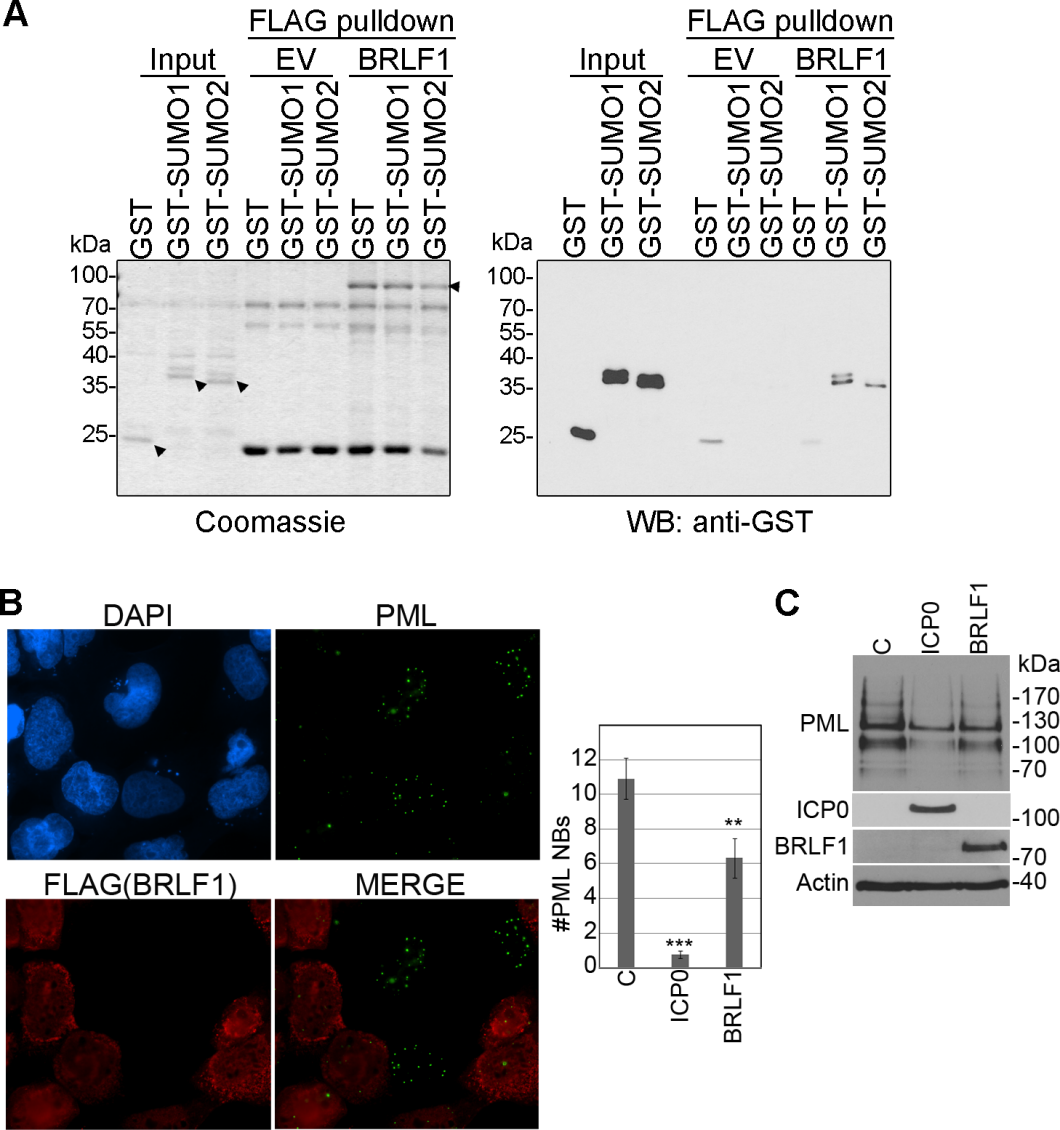
BRLF1 binds SUMO and induces loss of PML proteins and nuclear bodies. A. BRFL1-FLAG was purified from 293T cell lysate on anti-FLAG resin. Control resin loaded with the same 293T lysate lacking BRLF1 was generated as a control (EV). Resins were then incubated with *E*.*coli* extract containing equal amounts of GST, GST-SUMO1 or GST-SUMO2, washed and eluted in SDS buffer (FLAG pulldowns). The Coomassie gel on the left shows the BRLF1-FLAG and GST proteins used in the assay (arrowheads indicating full length proteins). The GST Western blot on the right shows the recovery of GST and GST-SUMOs. B. CNE2Z cells were transfected with plasmids expressing FLAG-tagged BRLF1, then, 24 hours later, fixed and stained for FLAG and PML. The number of PML NBs were counted in 50 FLAG-positive and 50 FLAG-negative cells on the same slide in two independent experiments, and average values are shown on the bar graph. The effect of ICP0 expression on the number of PML NBs in the CNE2Z cells is also shown on the graph as a positive control. p values (** = 0.001<*P* < 0.01; *** = *P* < 0.001) are shown. C. CNE2Z cells were transfected with plasmids expressing FLAG-tagged BRLF1, ICP0 or empty FLAG vector. 48 hours later, cells were lysed and immunoblotted for PML, ICP0, FLAG (BRLF1) and actin.

We next asked whether BRLF1 could bind SUMO. To this end, FLAG-BRFL1 was purified from 293T cells on anti-FLAG resin under high salt conditions (to limit protein interactions) and extensively washed (Fig 4A, left panel; BRLF1). Negative control resin was similarly generated using lysates from 293T cells with empty FLAG vector (Fig 4A, left panel; EV). GST-SUMO1, GST-SUMO2 or GST alone were generated in *Ecoli* ((Fig 4A, left panel) and equal amounts were applied to negative control or BRLF1-containing resin followed by extensive washing. Resin containing BRFL1, but not empty vector, retained GST-SUMO1 and GST-SUMO2 but not GST alone (Fig 4A, right panel), showing that BRLF1 can bind SUMO1 and SUMO2.

Since PML proteins are highly SUMO-modified, they are often a target of proteins that bind SUMO and disrupt/degrade SUMOylated proteins [15, 41–43]. Interestingly our previous screen of over 200 EBV, HSV1 and CMV proteins for ability to induce loss of PML NBs in U2OS cells identified BRLF1 as one of the top hits [33]. We further verified this property by examining the effect of BRLF1 on PML nuclear bodies and proteins in CNE2Z nasopharyngeal carcinoma cells (Fig 4B and 4C). BRLF1 consistently decreased the number and intensity of PML nuclear bodies and the level of PML proteins (although not as dramatically as ICP0 which is known to have multiple mechansims of targeting PML [44]). The results are consistent with the ability of BRLF1 to induce the loss of SUMO-modified proteins.

### SUMO induction activity of SM is conserved in homologues in other herpesviruses

**Fig 5:**
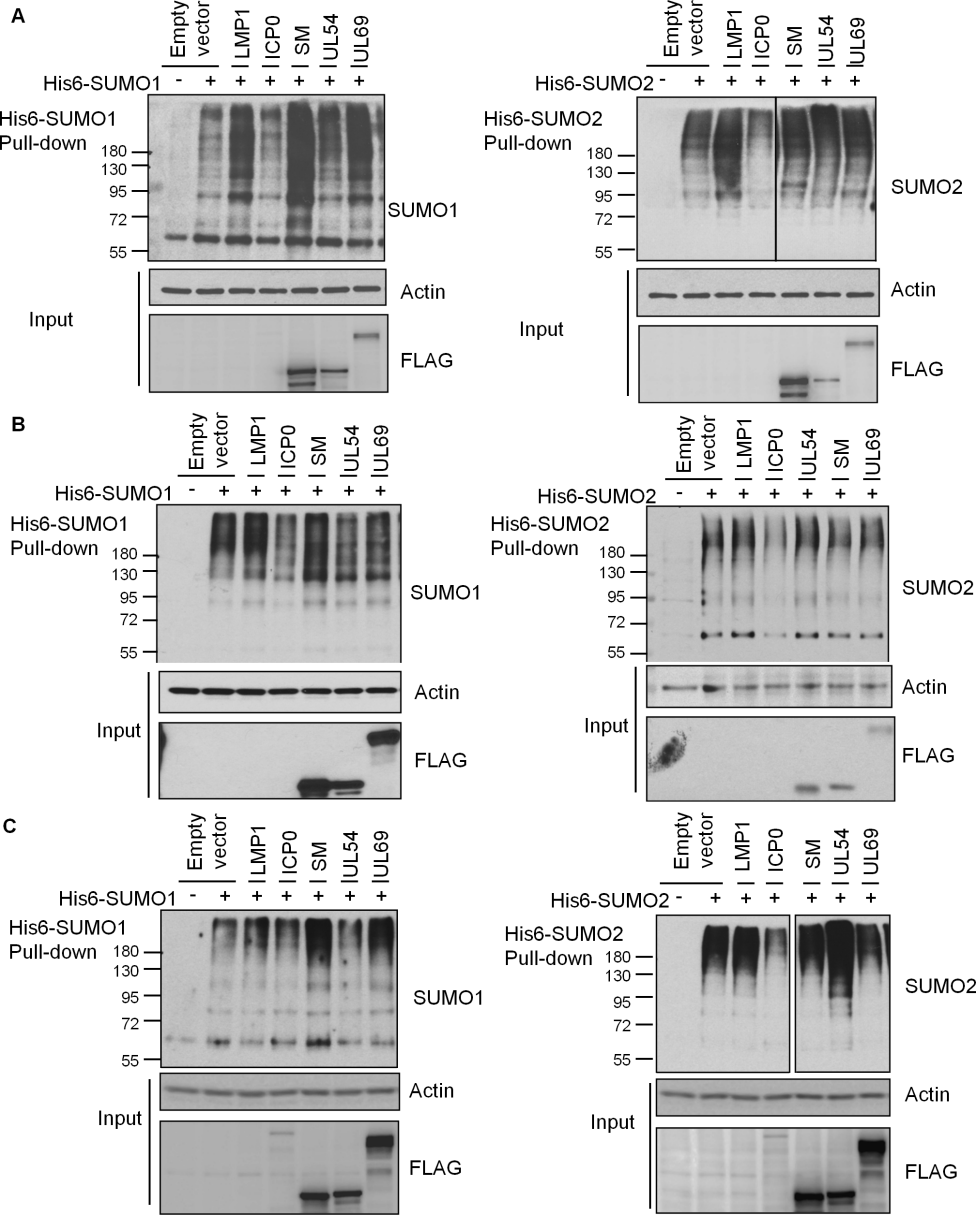
Effect of SM, UL54 and UL69 on global SUMO1 and SUMO2 modifications. 293T (A) and CNE2Z (C) cells were co-transfected with plasmids expressing His6-SUMO1 (left panels) or His6-SUMO2 (right panels) and either ICP0, LMP1, FLAG-SM, FLAG-UL54, FLAG-UL69 or empty vector control. HeLa cells (B) stably expressing His6-SUMO1 (left panels) or His6-SUMO2 (right panels) were transfected with plasmids expressing ICP0, LMP1, FLAG-SM, FLAG-UL54, FLAG-UL69 or empty vector control. His6-tagged proteins were recovered as in Fig 1 and immunoblotted for SUMO1 or SUMO2 as indicated. Samples of the input lysates were also immunoblotted for actin and FLAG.

Two of the proteins that upregulated SUMOylation in our screens, BMLF1 and SM (also called EB2) share common sequences, as BMLF1 is the C-terminal part of SM (Table 1, Fig 2, Fig 5). We focused our studies on SM since it is the functional protein. In assays in 293T cells detecting endogenous SUMO levels, SM expression was found to induce SUMO1 modifications without a noticeable effect on SUMO2 modifications (Fig 2). This was consistent with what we observed in the initial screen, where SUMO1 modifications were more obviously upregulated than SUMO2 modifications (Table 1). Interestingly, SM itself was efficiently modified by SUMO2 and less obviously by SUMO1 (S1 Fig). SM is known to have several roles in lytic EBV infection, including EBV mRNA export, splicing activation and transcriptional activation [45–49]. SM is conserved in all herpesviruses and its homologues in HSV1 (UL54 or ICP27) and CMV (UL69) have similar roles as SM in lytic infection [50, 51]. We reasoned that if SUMO induction by SM was important for viral infection, then this property would be conserved in the SM homologues in other herpesviruses. Therefore we compared the abilities of SM, UL54 and UL69 to induce SUMO1 and SUMO2 modifications in all three cell systems used for the initial screen (Fig 5). SM and UL69 were consistently found to upregulate SUMO1 modifications in all three cell lines, with little to no effect on SUMO2 modifications. Conversely, UL54 consistently increased SUMO2 modifications with little to no effect on SUMO1 modifications. Quantification from three independent experiments in each cell lines is shown in Table 2. These effects were not due to induction of SUMO or Ubc9 transcripts as neither SUMO1, SUMO2 nor Ubc9 mRNA levels were significantly affected by SM, UL54 or UL69 (S3 Fig). Since HSV1, CMV and EBV represent the three different subfamilies of herpesviruses (alpha, beta and gamma respectively), the results indicate that global induction of SUMOylation is an activity that is conserved in the SM family of proteins, although different family members have different specificities for SUMO1 vs SUMO2.

### SM, UL54 and UL69 bind SUMO and Ubc9

**Fig 6:**
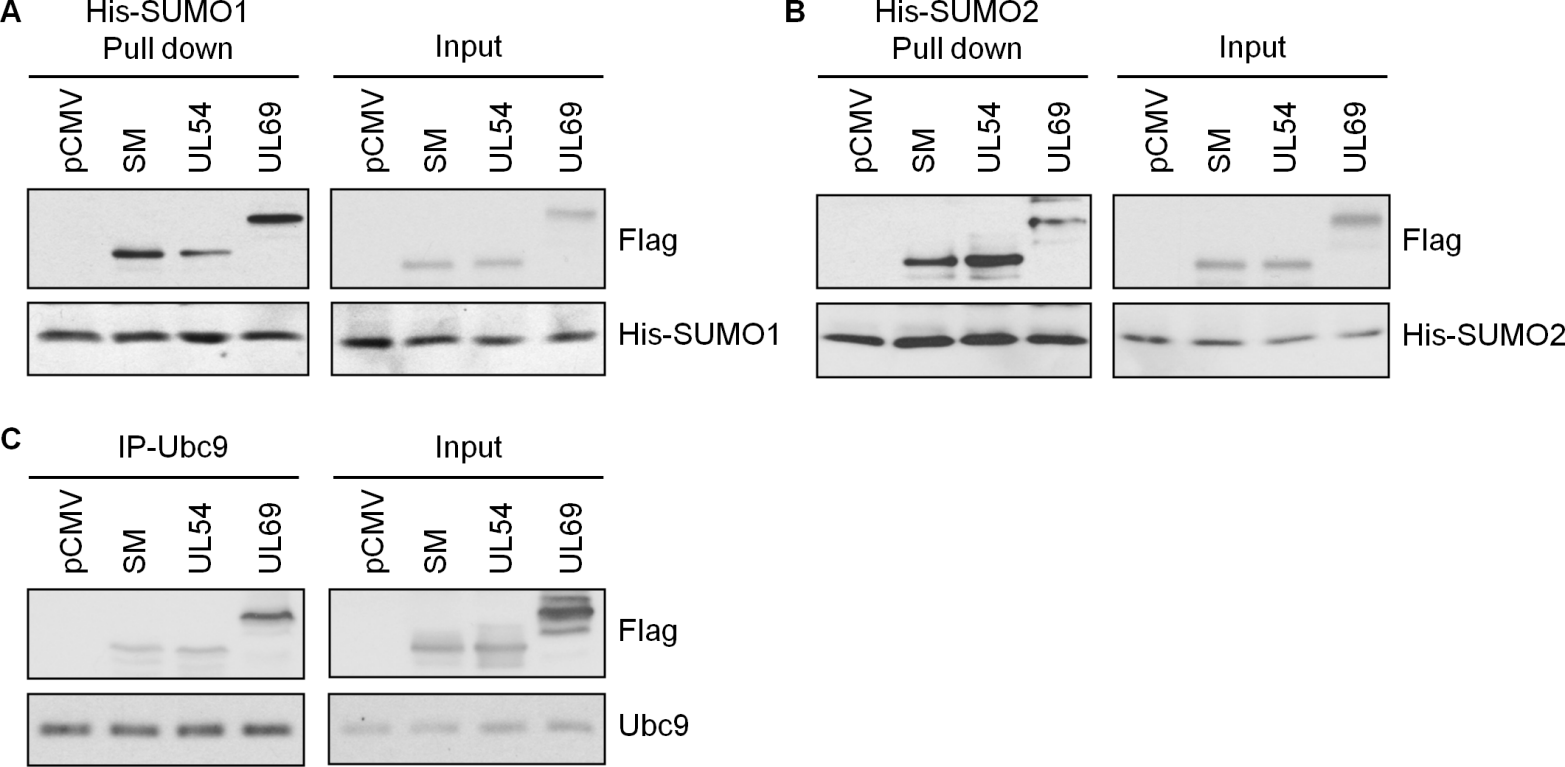
SM, UL54 and UL69 interact with SUMO1, SUMO2 and Ubc9 in human cells. A and B. HeLa cells containing integrated His6-SUMO1 (A) or His6-SUMO2 (B) were transfected with plasmids expressing FLAG-SM, FLAG-UL54, FLAG-UL69 or empty vector (pCMV). 36 hours later, His6-SUMO was recovered from cell lysates on metal chelating resin under nondenaturing conditions and immunoblotted for His and FLAG (left panels). 5% of the input lysate was also immunoblotted for His and FLAG (right panels). C. 293T cells were transfected with plasmids expressing FLAG-SM, FLAG-UL54, FLAG-UL69 or empty vector (pCMV). 36 hours later, immunoprecipitations were performed for endogenous Ubc9, followed by immunoblots with Ubc9 and FLAG antibody (left panels). Immunoblots were also performed on 5% of the input lysates (right panels). For all experiments, longer exposures were used for pulldowns/IPs than for inputs in order to provide optimum exposures to show differences between recoveries of different viral proteins in pulldowns and even levels of the viral proteins in inputs.

Proteins that directly affect SUMOylation typically bind to SUMO and/or the Ubc9 E2 SUMO-conjugating enzyme. Therefore to determine whether SM, UL54 and UL69 are acting directly on cellular SUMOylation, we first examined their association with SUMO1, SUMO2 and Ubc9 in human cells. To this end, HeLa cells containing integrated copies of His6-SUMO1 or His6-SUMO2 were transfected with FLAG-tagged SM, UL54 or UL69 followed by recovery of the His6-SUMOs on metal chelating resin under native conditions (Fig 6A and 6B). All three viral proteins were pulled down by SUMO1 and SUMO2 to varying degrees indicating that they interact with SUMO. However, SM and UL69 were more efficiently recovered with SUMO1 than UL54 (Fig 6A), and UL54 was more efficiently recovered with SUMO2 than SM and UL69 (Fig 6B). This specificity reflects the ability of these proteins to induce SUMO1 vs SUMO2 modifications, suggesting that the degree of SUMO binding influences their SUMOylation induction specificity.

**Fig 7:**
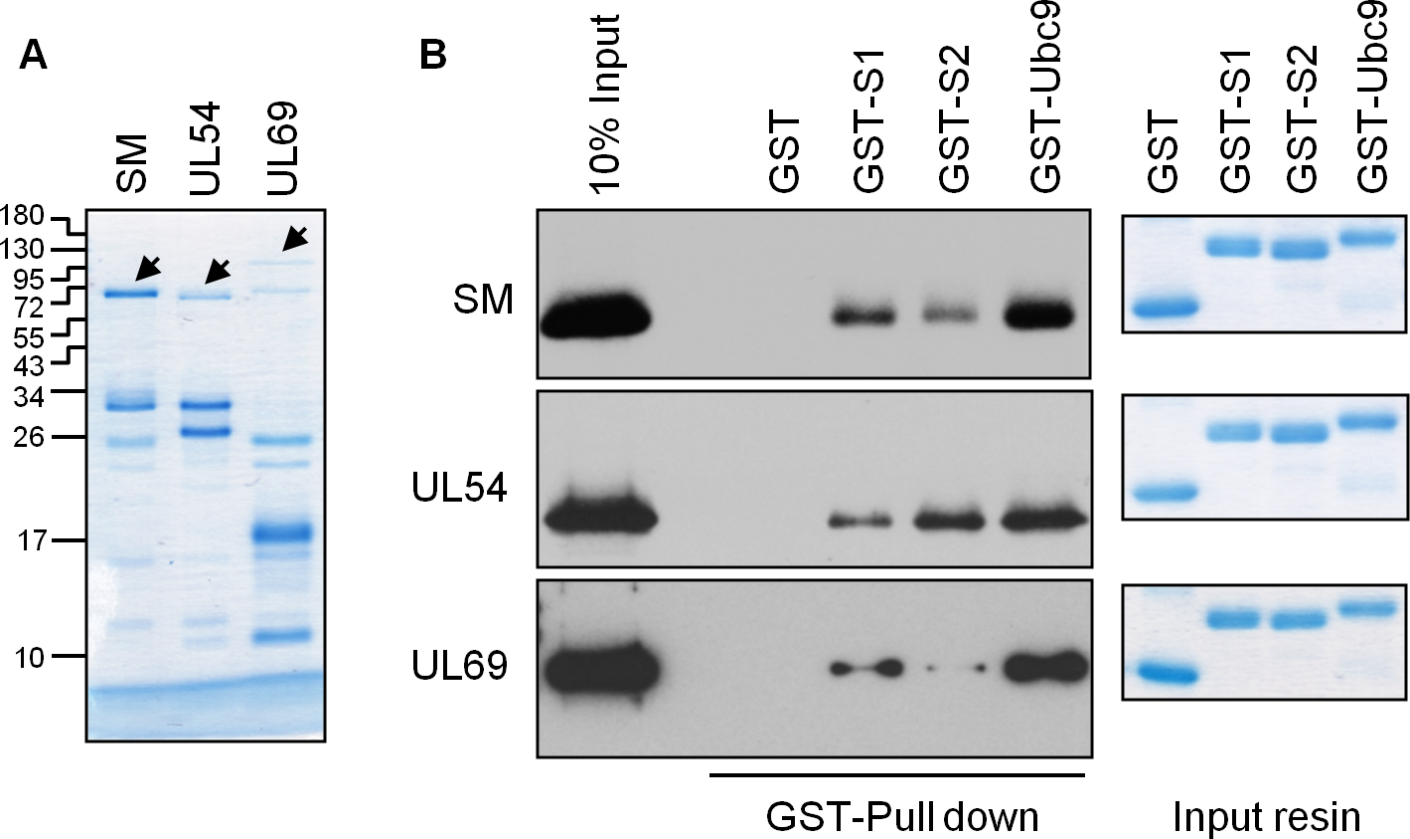
SM, UL54 and UL69 bind SUMO and Ubc9 in a purified system. A. His6-SM-3FLAG, His6-UL54-3FLAG and His6-UL69-3FLAG proteins were generated in *E*. *coli* and purified on nickel resin. Eluted protein was analyzed by SDS-PAGE and Coomassie staining. Arrowheads indicate the position of full length proteins. B. Equal amounts of the recombinant viral proteins in A (estimated to be ~1 μg of full length protein from Coomassie stained SDS-PAGs) were combined with equal amounts of GST-SUMO1, GST-SUMO2, GST-Ubc9 or GST control immobilized on glutathione resin. After washing, 10% of the resin was analysed by SDS-PAGE and Coomassie staining to assess the levels of GST proteins (right panels) and 90% was analysed by immunoblotting with anti-FLAG antibodies to assess recovery of the viral proteins.

To further investigate the SUMO specificities of the viral proteins and determine if SUMOs are bound directly, we expressed and partially purified FLAG-tagged SM, UL54 and UL69 from *E*.*coli* (Fig 7A) and used it in GST pull down assays with GST-tagged SUMO1 or SUMO2 (also generated in *E*.*coli*). All three viral proteins were retained on glutathione resin by GST-SUMO1 and GST-SUMO2 but not by GST alone (Fig 7B). However, once again the SUMO specificities varied among the viral proteins, with SM and UL69 being more efficiently bound by SUMO1 than SUMO2, and UL54 being more efficiently bound by SUMO2 than SUMO1. The results suggest that the ability to directly bind SUMO is an important factor in SUMOylation induction in cells.

We also asked whether SM, UL54 and UL69 interact with the E2 enzyme for SUMOylation, Ubc9. Immunoprecipitation of endogenous Ubc9 from 293T cells expressing SM, UL54 or UL69 showed that all three viral proteins were associated with Ubc9 (Fig 6C). We examined whether these associations were direct by using the *E*.*coli* purified viral proteins in GST pull down assays with GST-Ubc9 (Fig 7B). SM, UL54 and UL69 were all retained on glutathione resin by GST-Ubc9 but not by GST alone, showing that each can directly bind Ubc9.

### SM, UL54 and UL69 have E3 SUMO ligase activity *in vitro*

**Fig 8:**
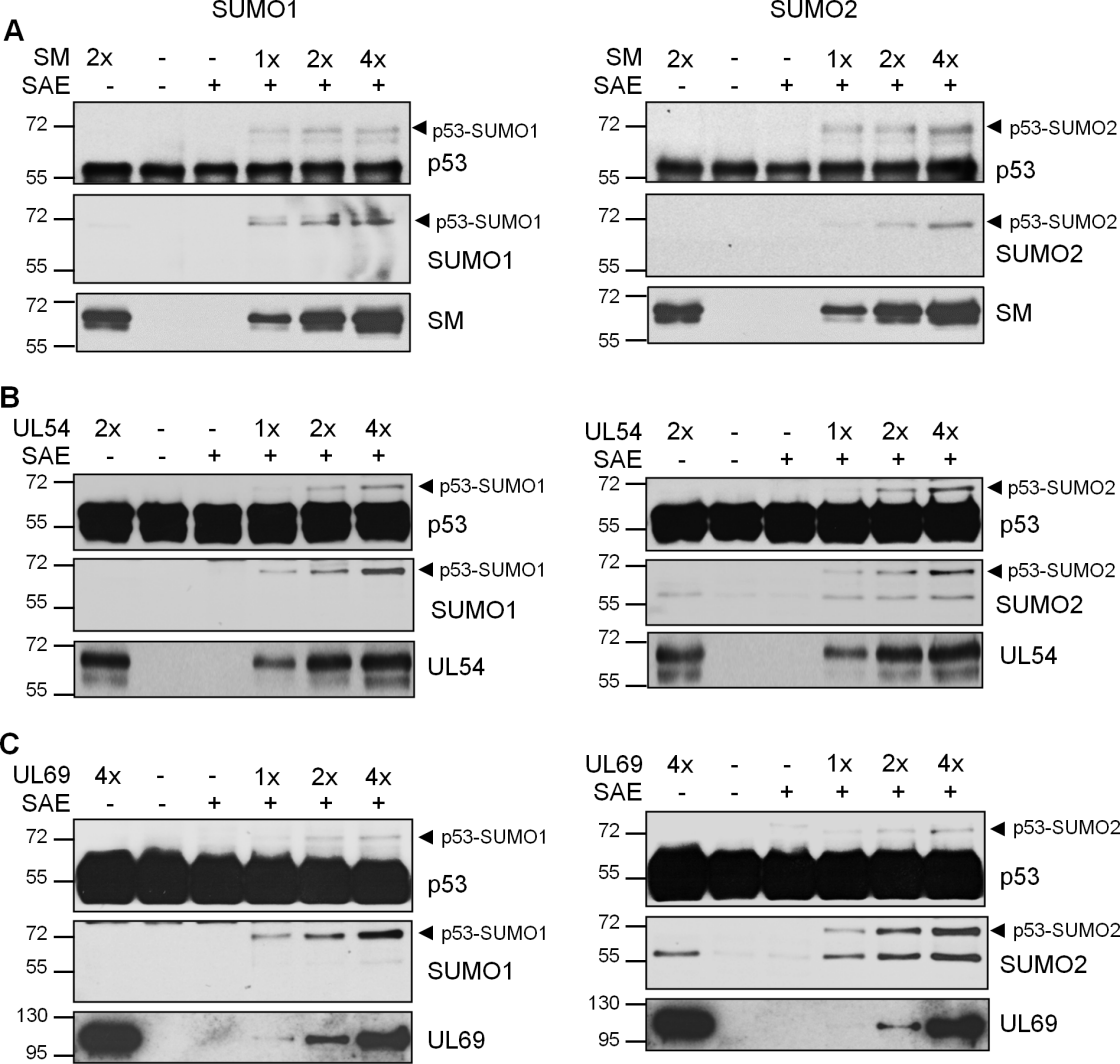
*In vitro* SUMO E3 ligase assays. *In vitro* SUMOylation assays were performed using Abcam SUMOylation Assay Kit with SUMO1 (left panels) or SUMO2 (right panels) and *E*.*coli* purified full length p53 as a substrate. Various amounts of His6-SM-3FLAG (A), His6-UL54-3FLAG (B) and His6-UL69-3FLAG (C), generated as in Fig 7A, were added to the indicated reactions where 1x is estimated to be 100 ng of full length protein. Negative controls lacking SAE are also shown. All lanes contained equal amounts of p53 substrate, SUMO and Ubc9. After 1.5 hrs reactions, Western blots were performed with antibodies against p53, SUMO1 (left panels), SUMO2 (right panels) and FLAG (to detect SM, UL54, UL69). The position of SUMO-modified p53 is indicated by the arrowheads.

The properties of SM, UL54 and UL69 in inducing global SUMOylation, binding directly to SUMO and binding directly to Ubc9 are consistent with SUMO E3 ligases. Therefore we investigated whether these viral proteins have SUMO E3 ligase activity in a purified *in vitro* system with the SUMO E1 (SAE) and E2 (Ubc9) enzymes, SUMO1 or SUMO2 and full length monomeric p53 as a substrate [52]. p53 was used as it is a well characterized substrate for SUMOylation that has previously been shown to require E3 ligases *in vitro* [10, 11, 13, 53]. All of the proteins in this assay (SAE, Ubc9, SUMO1, SUMO2, p53 and the viral proteins) were generated in *E*.*coli* to ensure that no contaminating SUMO E3 ligases are present. The addition of increasing amounts of purified SM (Fig 8A), UL54 (Fig 8B) or UL69 (Fig 8C) resulted in titratable induction of a shifted form of p53 consistent with mono-SUMOylated p53 (top panels). This shift was seen with both SUMO1 (left panels) and SUMO2 (right panels). Western blots for SUMO1 or SUMO2 confirmed that the shifted band was SUMO-modified p53 (middle panels). This band was not seen in assays using the highest amounts of the viral proteins in the absence of SAE, confirming that this SUMOylation was dependent on the E1 SUMO conjugating enzyme. Together the results indicate that SM, UL54 and UL69 can act as SUMO E3 ligases.

### SM, UL54 and UL69 can induce p53 SUMOylation in human cells

**Fig 9:**
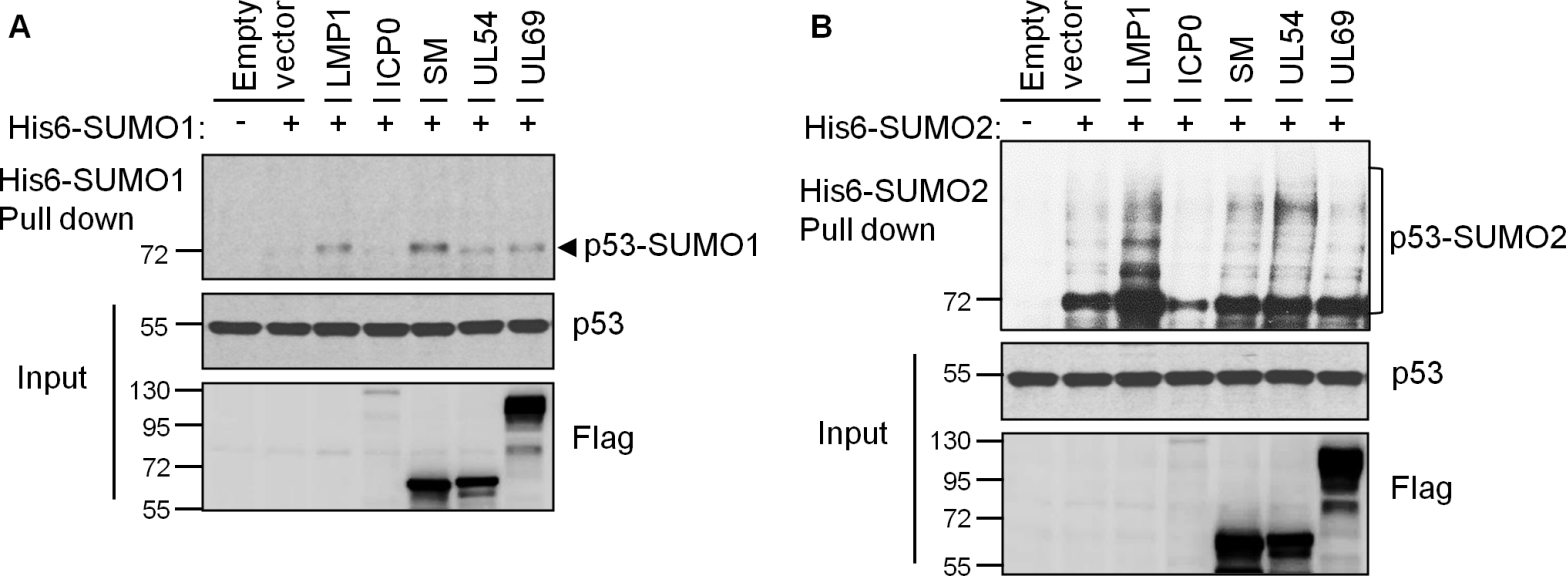
Induction of p53 SUMOylation by SM, UL54 and UL69 in cells. 293T cells were co-transfected with plasmids expressing His6-SUMO1 (A) or His6-SUMO2 (B) and plasmids expressing LMP1, ICP0, FLAG- SM, FLAG-UL54 or FLAG-UL69 or empty vector. His6-tagged proteins were recovered on metal chelating resin under denaturing conditions and immunoblotted for p53. Samples of the lysates (Input) were also immunoblotted for p53 and FLAG. Positions of SUMO1- or SUMO2-modified p53 are indicated.

Since we have shown that SM, UL54 and UL69 can all catalyze p53 SUMOylation *in vitro*, we investigated whether they also induced p53 SUMOylation in cells. To this end, 293T cells were co-transfected with constructs expression His6-SUMO1 or His6-SUMO2 and FLAG-tagged SM, UL54 or UL69. His-tagged proteins were then recovered on metal chelating resin under denaturing conditions and analysed by Western blotting using anti-p53 antibody. All three viral proteins were found to induce mono-SUMOylation of endogenous p53 by SUMO1, to a similar degree as the LMP1 positive control (Fig 9A). UL54 induced less SUMO1-modification of p53 than SM and UL69, consistent with the trend we observed for global SUMOylation. In SUMO2 experiments (Fig 9B), while the mono-SUMO2 p53 band (~70 KDa) was not obviously affected by SM, UL54 or UL69 as compared to the empty vector control, higher molecular weight SUMO2 products were evident with UL54 (and the LMP1 positive control). Together the results suggest that SM, UL54 and UL69 can all affect p53 SUMOylation, but with different SUMO1 vs SUMO2 specificities that reflect their effects on global SUMOylation and SUMO binding.

### Effect of SM depletion on SUMO1 profiles in EBV infection

**Fig 10:**
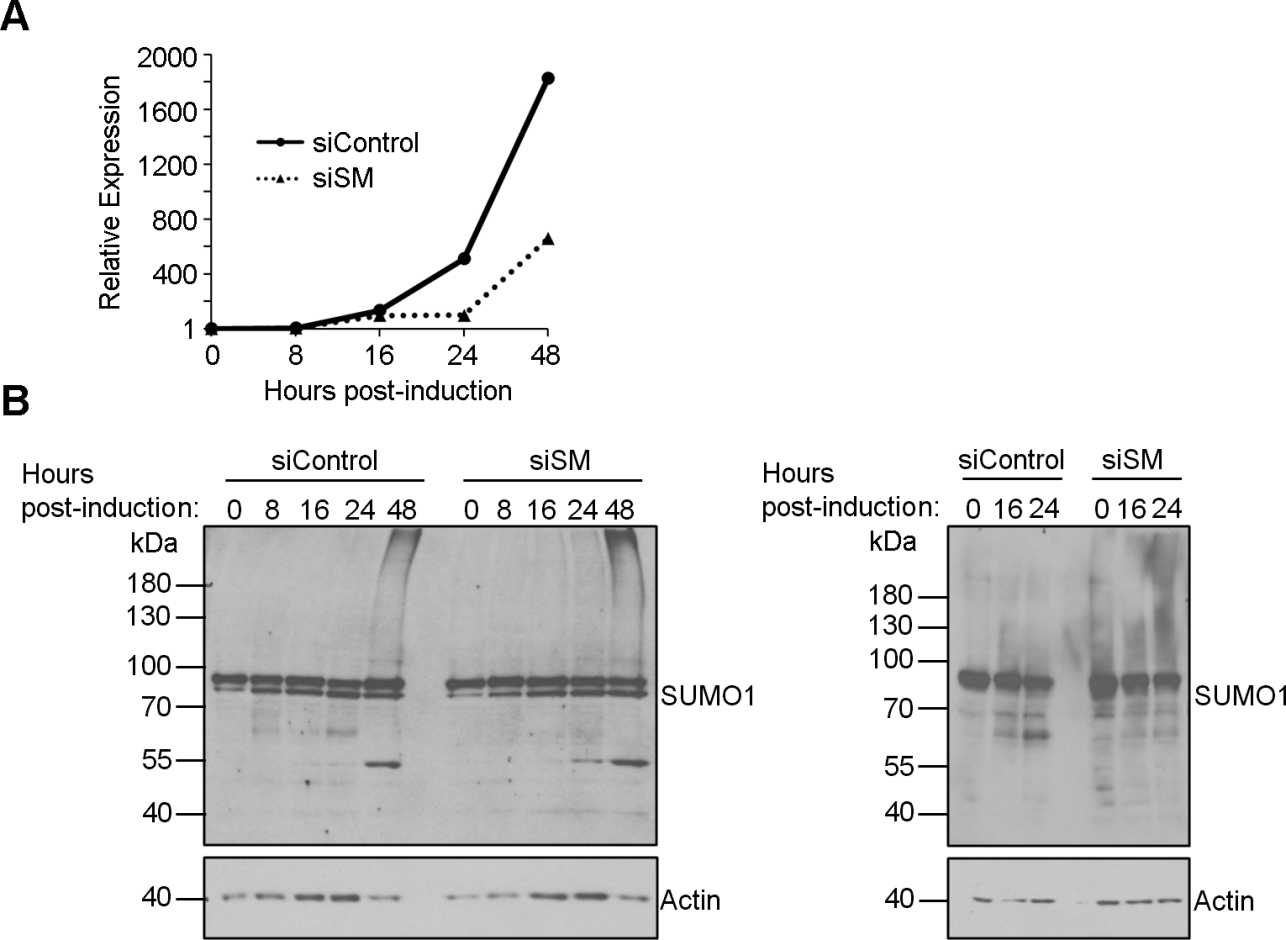
Silencing of SM in EBV lytic infection decreases some SUMO1 modifications. AGS-EBV cells were treated with siRNA against SM or negative control siRNA, then reactivated to the lytic cycle for the indicated times. A. SM mRNA was quantified by qRT-PCR. B. Cell lysates were analysed by Western blotting with antibodies against SUMO1 and actin (left panel). Pertinent time points were rerun with additional sample (right panel).

The fact that several EBV proteins can affect SUMOylation suggests a complicated interplay with SUMO pathways during EBV infection which would be best understood after identifying the specific targets of each of the EBV proteins. However, to begin to explore the effect of SM on SUMOylation in the context of EBV infection, we examined the cellular SUMO1 profile in EBV-positive gastric carcinoma cells (AGS-EBV) after lytic reactivation with and without SM depletion with SM-targeted siRNA. Quantification of SM transcripts showed that this gene was expressed by 16 hours post-reactivation and continued to increase in levels at 24 and 48 hour time points (Fig 10A). Pre-treatment with SM siRNA significantly decreased SM levels, particularly at 24 and 48 hour time points (Fig 10A). At the same time points, cell lysates were collected and analysed for SUMO1 profiles by Western blotting (Fig 10B). Two SUMO1-containing bands migrating at ~62 and 68 Kda were observed during the early stages of lytic infection but not at 48 hrs post induction (at which time greatly increased SUMO1 profiles suggest a stress response). These bands were reduced with SM depletion, most obviously at 24 hours post-induction when SM is accumulating in the cells. Note that these bands are too small to be any SUMO1-modified version of SM, which would migrate above the 72 Kda marker (although S2 Fig suggests there would be little SUMO1-modified SM). The results support the ability of SM to affect SUMOylation of some proteins in the context of EBV infection.

## Discussion

EBV manipulates many cellular pathways in order to promote infection and avoid host immune responses, and under some circumstances such alterations can lead to oncogenesis. Here we show that some EBV proteins can dramatically affect the SUMOylation of host proteins, providing an additional mechanism by which EBV can manipulate cells. We identified one EBV protein (BRLF1/Rta) that decreases the level of SUMOylated proteins and four distinct proteins that increase SUMOylated proteins. One of the latter proteins (SM) has characteristics of a SUMO E3 ligase, and this activity is conserved in SM homologues in HSV1 (UL54) and CMV (UL69) suggesting the importance of this activity for herpesvirus infections.

Our screen using overexpressed EBV proteins was designed so that viral proteins with the ability to affect SUMO pathways would manifest as a global SUMOylation phenotype, even though in the context of infection they may only affect a subset of SUMOylated proteins. Herpesviruses in lytic infection modulate a wide variety of cellular processes that involve SUMOylation, the best studied being the disruption of PML NBs by viral SUMO-targeted ubiquitin ligases that degrade PML proteins or SUMO-interacting proteins that disrupt PML protein interactions [15, 19, 54–57]. Indeed, the SUMO downregulator that we identified here (BRLF1) was also shown to disrupt PML NBs. Additional SUMO-regulated processes controlled by herpesviruses include interference with cell cycle progression, resulting in G1/S arrest, and inhibition of DNA damage responses [58–64]. Our previous screens have identified BGLF2 and BMRF1 as contributing to G1/S arrest [28] and BMRF1 as an inhibitor of DNA damage response [65]. We do not yet know the mechanism of these effects, but the fact that both proteins were found to upregulate SUMOylation suggests that they may be affecting cell cycle progression and the DDR by increasing SUMOylation of some cellular proteins.

The only EBV protein that we found to globally decrease SUMOylation was BRLF1 (also called Rta); an immediate early protein conserved in γ-herpesviruses and known to function as a transcriptional activator. Interestingly, the BRLF1 homologues (Rta) in KSHV and murine γHV68 were both shown to have ubiquitin ligase activity [18, 40] and KSHV Rta was later shown to be a SUMO-targeted ubiquitin ligase (STUbL) [15]. Like these Rta proteins, the protein loss we observed with BRLF1 was proteasomal dependent, suggesting that induction of protein degradation is a conserved function of the Rta proteins. While the ability of γHV68 Rta to bind SUMO has not been reported, it is known to degrade the RelA subunit of NF-κB which is highly SUMO-modified by PIAS3, raising the possibility that SUMOylation of RelA is part of the targeting mechanism by Rta [18, 66]. KSHV Rta was shown to contain SUMO interacting motifs that bind SUMO and to induce degradation of the highly SUMOylated PML proteins [15]. In addition, the STUbL activity of KSHV Rta was shown to be required for transcriptional activation, suggesting that removal of suppressive SUMOylated proteins promotes transcription [15]. Similarly, we found that EBV Rta binds SUMO and induces loss of PML NBs and proteins ([33] and Fig 4). In addition, EBV Rta is SUMO modified and this modification has been found to increase its transactivation activity [33, 67, 68]. However, despite the many similarities between KSHV and EBV Rta, we could not find evidence of ubiquitin ligase activity in EBV Rta, even though this activity was readily apparent in the same assay with KSHV Rta (Fig 3C). This is likely due to the low sequence conservation between these proteins, including the lack of catalytic cysteines which are conserved in KSHV and γHV68 Rta [18]. Interestingly, a KSHV Rta mutant lacking ubiquitin ligase activity still induces the degradation of PML proteins suggesting that it has two mechanisms of degrading SUMOylated proteins [15]. This second (unknown) mechanism of degradation of SUMOylated proteins may be conserved in EBV Rta.

Our screen identified four distinct EBV proteins that globally upregulated SUMOylation. (SM, BGLF2, BMRF1, BVRF2). None of these proteins have been previously shown to impact SUMO pathways, although both BMRF1 and BVRF2 were shown to be SUMO-modified [32, 69]. Since global SUMOylation patterns can be caused by stress responses, it is possible that some of these proteins are eliciting a stress response [70]. Little is known about BVRF2 other than its conserved role as a scaffold protease [71]. BGLF2 is a viral tegument protein that has been found to induce p21 and interfere with G1/S cell cycle progression [28] and also to induce BZLF1 expression and the AP-1 signalling pathway through p38 and c-Jun N-terminal kinases activation [72, 73]. BMRF1 belongs to the family of DNA polymerase processivity factors that are conserved in all herpesviruses [74]. However, BMRF1 also has additional roles in transcriptional activation [75–77], cell cycle progression [28] and in inhibiting the DNA damage response to double stranded DNA breaks [34]. It will be interesting to determine how the multiple roles of BGLF2 and BMRF1 relate to their ability to upregulate SUMOylation.

One of the EBV proteins identified in our screens that globally upregulated SUMOylation was the SM (or EB2) protein that is conserved in other herpesviruses. SM and its homologues in HSV1 (UL54 or ICP27) and CMV (UL69) have conserved functions in RNA binding, splicing modulation and the export and translation of viral mRNA [45, 46, 48–51, 78]. In addition, several studies have identified roles for HSV1 UL54 in cell signalling and apoptosis [51, 79–81]. Our findings that SM, UL54 and UL69 induce SUMOylation suggests that the cellular effects of these proteins may be more extensive than is currently known. In addition, SUMOylation might play roles in the known functions of these proteins. While the role of SUMOylation in RNA splicing and mRNA transport has not been extensively studied, heterogeneous nuclear RNA binding proteins (hnRNPs), which have multiple roles in RNA splicing stabilization and export, are highly SUMOylated by both SUMO1 and SUMO2/3 [82–84]. In addition, the RanBP2/Nup358 component of the nuclear pore complex, which plays roles in mRNA export and translation, is a SUMO E3 ligase [36, 85, 86].

Our data suggest that SM, UL54 and UL69 are SUMO E3 ligases. These proteins not only upregulated SUMOylation in multiple cell systems, but bound directly to SUMO and Ubc9, as expected of SUMO E3 ligases. In addition, in a purified system using *E*.*coli* generated proteins, SM, UL54 and UL69 all induced SUMOylation of p53 in conjunction with Ubc9 and SAE. In cells, the three viral proteins varied in their abilities to induce SUMO1 vs SUMO2 modifications, with SM and UL69 preferentially inducing SUMO1 modifications and UL54 preferentially inducing SUMO2 modifications. This preference was also reflected in SUMO binding assays, where both immunoprecipitations from cells and *in vitro* SUMO binding assays showed that SM and UL69 preferentially bound SUMO1, while UL54 preferentially bound SUMO2. Preferences for SUMO1 or SUMO2/3 have been previously reported for some other SUMO binding proteins. For example, the KSHV K-bZIP and cellular ZNF451 SUMO E3 ligases have been shown to have specificity for SUMO2/3 over SUMO1, due to the presence of SIMs that preferentially bind SUMO2/3 over SUMO1 [13, 87, 88]. In contrast ORF61 of Varicella-Zoster virus (orthologue of the HSV1 ICP0 STUbL) preferentially binds SUMO1 [21]. SIMs can vary considerably in sequence, making them difficult to accurately predict by sequence analysis [89], and hence the SIMS in SM, UL54 and UL69 have yet to be determined.

SUMO E3 ligases are also difficult to predict since their sequence and structures vary considerably. The PIAS family of SUMO E3 ligases contain modified RING domains similar to RING-type ubiquitin ligases, however other SUMO E3 ligases, such as RANBP2 and K-bZIP, lack conserved catalytic domains [89, 90]. Zinc fingers have been found to be an important component of some E3 SUMO ligases, for example RANBP2 contains eight tandem zinc fingers and the ZNF451 family of SUMO E3 ligases contain 12 C2H2 zinc-finger domains [88, 91]. Interestingly, the structure of the C-terminal domain of ICP27 (UL54) revealed a novel CHCC-type zinc finger in which the zinc coordinating residues are conserved in UL54 homologues in other herpesviruses, including UL69 and SM [92, 93]. The conservation of this zinc-binding domain, and the presence of similar zinc binding domains in cellular SUMO E3 ligases, suggests that it might be an important component of the SUMO E3 ligase activity of SM, UL54 and UL69.

We have shown that p53 is one cellular protein that can be SUMOylated by SM, UL54 and UL69. p53 is known to be modified by either SUMO1 or SUMO2/3 at K386 [94]. The consequences of these modifications are a matter of debate, as there are reports that these modifications increase transcriptional activation by p53 and other reports that they decrease p53 activity [13, 94–97]. We showed that SM, UL54 and UL69 are all capable of inducing SUMO1 and SUMO2 modifications of p53 under *in vitro* conditions. In cells we also observed increased SUMOylation of p53 by these viral proteins, although SM and UL69 preferentially increased SUMO1 modification while UL54 increased SUMO2 modifications. This pattern parallels the global SUMOylation effects of these viral proteins. Whether or not p53 is an intended SUMOylation target of these viral proteins or whether other cellular proteins are preferential targets remains to be determined. Although SM, UL54 and UL69 globally upregulate SUMOylation under our assays conditions, we imagine that these proteins have preferred substrates during infection. Identifying these substrates will provide important insights into the functions and mechanisms of action of these proteins.

Our results build on the growing body of literature that manipulation of host SUMO pathways is important for lytic infection by herpesviruses. KSHV is known to express both a STUbL (Rta) and a SUMO2/3-specific SUMO E3 ligase (K-bZIP) in lytic infection, and SUMO2/3 modifications in general have been shown to suppress KSHV reactivation and expression of KSHV lytic genes [13, 15, 98, 99]. HSV1 is also known to encode a well-studied STUbL (ICP0)[16]. Our study provides the first identification of SUMO E3 ligases in HSV1, CMV and EBV, and also identifies EBV BRLF1 as a negative regulator of SUMOylation. To our knowledge, these are the first reports of EBV lytic proteins that globally affect SUMOylation. Previous studies on EBV and SUMOylation pathways have shown that LMP1 promotes latency by upregulating SUMOylation in EBV latent infection [30, 31], and that one of the EBV miRNAs expressed in lytic infection can promote SUMOylation by downregulating RNF4 [32]. In addition, several EBV proteins have been found to be SUMO modified, suggesting that their activities can be regulated by SUMOylation [32, 33, 39, 69]. It will be interesting to determine how the interplay between viral proteins that increase and decrease SUMOylation contribute to herpesvirus infections and why UL54 preferentially induces SUMO2 modifications while its homologues in EBV and CMV induce SUMO1 modifications.

## Materials and methods

### Plasmids

The EBV expression library was generated in pMZS3F for expression in mammalian cells, as previously described [28], such that proteins are expressed fused to a C-terminal calmodulin binding peptide and triple FLAG epitope. EBV SM in pcDNA3 (a generous gift from Sankar Swaminathan) and EBV BRLF1 in pMZS3F were subcloned between the Sal I and Xba I sites in pCMV3FC, and HSV-1 UL54/ICP27 and CMV UL69 in pMZS3F were subcloned between the Xho I and Xba I sites in pCMV3FC, to generate proteins with C-terminal triple FLAG tags. Plasmids encoding EBV HA-tagged LMP1 (a gift from Nancy Raab-Traub) [100], HSV-1 ICP0 (pCI-110; a gift from Roger Everett)[101] and human His6-SUMO1 and His6-SUMO2 (in pcDNA3; gifts from Ronald T. Hay) [38, 102] for expression in mammalian cells have been previously described. For expression in *E*. *coli*, SM, UL54/ICP27 and UL69 coding sequences were excised from pCMV3FC using the restriction enzymes indicated above and were ligated into the corresponding sites of a modified pET15b with a multicloning site. To generate the modified pET15b, the Xba I site was mutated in pET15b then oligos (5'- CCA TGG GCA GCA GCC ATC ATC ATC ATC ATC ACA GCA GCG GCC TCG AGG CTA GCG TCG ACG GTA CCT CTA GAG ACG TAG CGG CCG CGG CGG ATC C -3') containing a multicloning sequence (Xho I, Nhe I, Sal I, KpnI, Xba I, Not I) were inserted between the Nco I and Bam HI sites of pET15b downstream of the hexahistidine tag. Oligos encoding a triple FLAG tag were then inserted between the Not I and Bam HI sites so that recombinant proteins contain N-terminal His6 and C-terminal 3FLAG tags. pGEX2T-SUMO1, pGEX2T-SUMO2 and pGEX2T-Ubc9 were kindly provided by Ronald T. Hay [103]. pcDNA4/TO plasmid expressing KSHV Rta (ORF50) with C-terminal Strep tag was a gift from Britt Glausinger and is described in Davis et al 2015[104]. The plasmid expressing His-Myc-ubiquitin (pHis-Myc-Ub) was a gift from Filippo Giancotti [105]

### Cell lines and transfections

HeLa cells (cervical carcinoma) containing integrated His6-SUMO1 or His6-SUMO2 (kindly provided by Ronald T. Hay; [106]) and 293T cells (embryonic kidney; ATCC) were cultured in DMEM with 10% FBS. CNE2Z cells (EBV-negative nasopharyngeal carcinoma [107]; a gift from Fei-Fei Liu) were cultured in α-MEM with 10% FBS. AGS-EBVcells (EBV- positive gastric carcinoma [108]; a gift from Lindsay Hutt-Fletcher) were grown in RPMI with 10% FBS. All cells were plated 24 hrs before transfection and transfected at a confluency of 70–80% using PolyJet (FroggaBio) or Lipofectamine 2000 (Life Technologies) or linear polyethylenimine (PEI; Polyscience Inc. catalogue number 23966), as suggested by the manufacturer.

### Western blotting and antibodies

Protein fractions were separated by SDS-PAGE (8% to 15% depending on the experiment) and transferred onto nitrocellulose. Membranes were blocked with 1% BSA in PBS-Tween 0.1% (PBS-T) for 1 h, followed by incubation with primary antibodies in blocking buffer overnight at 4°C. Membranes were washed three times with PBS-T for 10 min and then incubated with secondary antibodies conjugated to horseradish peroxidase in blocking buffer for 1 h. Membranes were washed four times with PBS-T for 15 min, and signals were detected by enhanced chemiluminescence (Santa Cruz sc-2048 or Biorad Clarity 1705061). Antibodies to SUMO1 (FL-101, 1:1000 dilution), SUMO2 (FL-103, 1:2000 dilution), Ubc9 (H-81, 1:500 dilution), His (H-15, 1:500 dilution), p53 (DO-1, 1:5000 dilution), myc (sc-40; 1:2000 dilution) and actin (C-11, 1:2000 dilution) were from Santa Cruz. Antibodies to PML (A301-167A, 1:2000 dilution), GST (A190-122A; 1:10,000) and FLAG (S190-102, 1:10000 dilution) were from Bethyl. Anti-FLAG (F1804, 1:10000 dilution) was from SIGMA and anti-ICP0 (H1A027, 1:5000 dilution) was from Virusys. RNF4 antibody (a kind gift from Ronald T. Hay; [109]) and was used at 1:5000 dilution.

### Global SUMOylation screen

293T and CNE2Z cells in 6 well dishes were co-transfected with plasmids (0.5 μg each) expressing His6-SUMO1 or His6-SUMO2 and FLAG-tagged viral proteins (in pMZS3F or pCMV3FC) or LMP1 (positive control for SUMO upregulation) or ICP0 (positive control for SUMO downregulation) or empty plasmid negative control. HeLa cells containing integrated His6-SUMO1 or His6-SUMO2 were similarly transfected with 0.5 μg of plasmids expressing FLAG-tagged viral proteins, ICP0 or LMP1 or empty plasmid control. His6-tagged SUMO conjugates were purified under denaturing conditions essentially as previously described [106]. Briefly, approximately 2 x 10^6^ cells were harvested 36 hrs post transfection. 10% of the cells were lysed in 2X SDS loading buffer (60 mM Tris.HCl pH 6.8, 1% SDS, 100 mM DTT, 5% glycerol) to provide the input sample. 90% of the cells were resuspended in 0.5 ml lysis buffer G (6 M guanidine hydrochloride, 10 mM Tris, 100 mM sodium phosphate, pH 8.0) and incubated on ice 20 min. Lysates were passed through a 30G needle five times. Then, lysates were added 50 μl of TALON Metal Affinity Resin (Clontech) previously equilibrated with lysis buffer, and incubated 3 hrs at room temperature with end-over-end rotation. The resin was washed four times with 1 ml of wash buffer U (8 M urea, 10 mM Tris, 100 mM sodium phosphate, pH 8.0) and proteins were eluted in 2X SDS loading buffer. Inputs and purified fractions were analyzed by Western blotting with SUMO1, SUMO2, FLAG and actin antibodies.

### RNF4 silencing

For SUMOylation experiments involving RNF4 silencing, CNE2Z were plated in a 6-well dish at 10% confluency and transfected with either 40 pmoles of Stealth siRNF4 (Invitrogen) or Qiagen Allstars control siRNA with Lipofectamine 2000 (ThermoFisher Scientific) according to the manufacturer’s instructions. The transfection was repeated after 24 hours. 9 hours later the cells were transfected (using PEI) with1 μg of pCMV3FC, pCI-110 (expressing ICP0) or pCMV3FC-BRLF1 and 1 μg of plasmids expressing His6-SUMO1 or His6-SUMO2. Cells were harvested 39 hours later and lysed in 150 μl of 8 M urea buffer (8 M urea, 20 mM Tris pH 8, 100 mM NaCl, protease inhibitor cocktail (P8849; Sigma)). Lysates were sonicated and clarified by centrifugation. 360 μg of clarified lysate was incubated with 50 μl equilibrated TALON resin for 2 hours at RT with mixing, followed by washing and elution as above. Elutions were analysed by 8% SDS-PAGE and Western blotting for SUMO1 or SUMO2. 30 μg of each clarified lysate was analysed by 12% SDS-PAGE and Western blotting for RNF4, ICP0, FLAG (BRLF1) and actin (Thermofisher).

### Effect of MG132 on endogenous SUMOylation by BRLF1

293T cells in 6 well dishes were transfected with 2 μg plasmid expressing ICP0 or FLAG-tagged BRLF1 using PEI according to the manufacturer’s protocol. For one set of samples, MG132 (Sigma) was added to 10 μM 24 hours post-transfection and 10 hours prior to harvesting. All samples were harvested 34 hours post-transfection and lysed in 9 M urea, 10 mM Tris pH 6.8 with sonication. 40 μg of clarified lysates were analysed on 8% SDS-PAGs followed by Western blotting with antibodies against SUMO1, SUMO2, FLAG, ICP0 and actin.

### Ubiquitylation assay

CNE2Z or 293T in 6 well dishes were transfected with PEI with 1 μg pCMV-3FC, pCI-110 (expressing ICP0), pcDNA4/TO expressing Strep-tagged KSHV Rta or pCMV3FC-BRLF1 and 1 μg of plasmid expressing His-Myc-ubiquitin [105]. 24 hours later, MG132 (Cell Signalling, 2194S) was added to 10 μM for 10 hours. Cells were harvested and lysed in 150 μl (CNE2Z) or 300 μl (293T) 8M urea buffer (8M urea, 20 mM Tris pH 8,100 mM NaCl, protease inhibitor cocktail (Sigma P8849)). Lysates were sonicated and centrifuged to clarify. 50 μg (CNE2Z) or 160 μg (293T) of lysate was added to 50 μl TALON resin (prewashed with 8M urea buffer), followed by incubation, washing and elution as described above for SUMOylation screen. 30 μl of elutions and 30 μg of clarified lysates were analysed by Western blotting using anti-myc antibody. Input lysate were also probed with antibodies against FLAG (BRLF1), ICP0 and Strep (KSHV Rta).

### PML nuclear bodies (NB) and protein analyses

Immunofluorescence microscopy analysis was performed as described previously [45]. Briefly, CNE2Z cells on cover slips were transfected with plasmids expressing ICP0 (pCI-110) or FLAG-tagged BRLF1 (in pCMV3FC), fixed 24 hrs post transfection and stained with antibodies against PML and ICP0 or FLAG. PML NBs were counted in 50 cells for each sample in two independent experiments. For PML Western blots, cells were transfected as described above and, 48 hrs post transfection, cells were lysed in 9M urea buffer (9M urea, 10 mM Tris pH 6.8). 40 μg of clarified lysates were loaded onto 10% SDS-PAGE, transferred onto nitrocellulose and analyzed by Western blotting with PML, ICP0 and FLAG antibodies.

### Analysis of endogenous SUMOylation

293T cells in 12 well plates were transfected with 0.5 μg plasmid expressing LMP1, ICP0 or the indicated FLAG-tagged EBV protein. 36 hrs later, 1 x 10^6^ 293T cells were lysed in passive lysis buffer (Promega E194A). 50 μg of clarified lysates were loaded onto 8% SDS-PAGE, transferred to nitrocellulose and analyzed by Western blotting with SUMO1, SUMO2, FLAG and actin antibodies.

### SUMO1, SUMO2 and Ubc9 interactions in human cells

To evaluate the interaction of SM, UL54, and UL69 with SUMO proteins, HeLa cells containing integrated His6-SUMO1 or His6-SUMO2 in 6 cm dishes were transfected with 2.5 μg of pCMV3FC plasmids expressing FLAG-tagged SM, UL54 or UL69 or with pCMV3FC alone. 36 hrs later, cells were harvested and lysed in RIPA buffer without EDTA [50 mM Tris pH 8.0, 200 mM NaCl, 1.0% (v/v) NP-40, 0.5% (w/v) sodium deoxycholate, protease inhibitor cocktail SIGMA P8340]. Lysates (1 mg) were subjected to His-pull down using 100 μl of Ni-NTA agarose (Qiagen) for 2 hrs at 4°C with mixing, and beads were then washed four times with 1 ml of RIPA buffer. Proteins were eluted in 2X SDS loading buffer and analyzed by Western blotting with antibodies against FLAG and His. To evaluate the interaction of SM, UL54, and UL69 with Ubc9, 293T cells were transfected as described above, and 36 hrs post-transfection cells were lysed in RIPA buffer. 2 mg of lysate was incubated overnight at 4°C with 25 μg of agarose-conjugated Ubc9 antibody (C-12; Santa Cruz), with mixing, then beads were washed four times with 1 ml of RIPA buffer. Proteins were eluted in 2X SDS loading buffer and analyzed by Western blotting with FLAG and Ubc9 antibodies.

### Protein purification from *E*.*coli* for *in vitro* assays

Recombinant His6-SM-3FLAG, His6-UL54-3FLAG and His6-UL69-3FLAG proteins were generated in *E*. *coli* for *in vitro* assays. BL21-pLysS *E*. *coli* containing pET15b-SM-3FLAG, pET15b-UL54-3FLAG or pET15b-UL69-3FLAG were grown in Luria broth (LB) to OD_580_ 0.6 then protein expression was induced with 1 mM IPTG overnight at 18°C. Bacteria from 1 L of culture were resuspended in 20 ml of binding buffer (50 mM sodium phosphate, 300 mM NaCl, 5 mM imidazole, protease inhibitor cocktail, pH 7.8) and lysed with 3 rounds of sonication for 20 sec each. Lysates were clarified by centrifugation at 10000 x g for 20 min at 4°C and then incubated with 400 μl of Ni-NTA agarose (Qiagen) for 1 h at 4°C with mixing. Agarose was washed 4 times with 2 ml washing buffer (50 mM sodium phosphate, 300 mM NaCl, 20 mM imidazole, protease inhibitor cocktail, pH7.6), then transferred into a gravity-flow column and washed once more with 2 ml washing buffer. Proteins were eluted with 1 ml of elution buffer (50 mM sodium phosphate, 300 mM NaCl, 200 mM imidazole, protease inhibitor cocktail, pH7.4) and collected in 200 μl fractions. Hexahistidine-tagged full length p53 with L344P point mutation (rendering it monomeric) was expressed and purified from *E*.*coli* as described previously [52]. GST, GST-SUMO1, GST-SUMO2 and GST-Ubc9 were generated by standard methods. Briefly, DH5α *E*. *coli* containing pGEX-2T expression plasmids were grown in LB to OD_580_ 0.6, then protein expression was induced with 1 mM IPTG for 2 hrs at 37°C. Bacteria from 1 L of culture were resuspended in 20 ml of PBS supplemented with 1% Triton X-100 and 1 mM PMSF, and lysed with 3 rounds of sonication for 20 sec each. Lysates were clarified by centrifugation at 10000 x g for 20 min at 4°C and then incubated with 500 μl of Glutathione Sepharose 4B (GE life sciences) for 2 hrs at 4°C with mixing. Resin was washed 4 times with 2 ml PBS-TritonX100-PMSF, then resuspended in 0.5 ml PBS with 1 mM PMSF and stored at -80°C.

### In vitro binding assays with SM, UL54, UL69

GST-pull down assays were performed to evaluate the interaction of purified, recombinant SM, UL54 and UL69 with GST-tagged SUMO1, SUMO2 and Ubc9. To this end, GST-tagged proteins (or GST alone) bound to glutathione resin (described above) were blocked with 2% BSA in PBS for 2 hrs at 4°C then levels of the GST proteins were evaluated by SDS-PAGE and Coomassie blue staining. Equal amounts of GST-SUMO1, GST-SUMO2, GST-Ubc9 or GST control bound to resin (corresponding to ~2 μg of full length protein) were combined with equal amounts (~1 μg as estimated from Coomassie stained SDS-PAGs) of full-length recombinant His6-SM-3FLAG, His6-UL54-3FLAG or His6-UL69-3FLAG (partially purified from *E*.*coli* as described above) in 150 μl binding buffer (20 mM Tris pH 8.0, 200 mM NaCl, 0.2 mM EDTA, 10% glycerol, 0.1% Triton X-100, protease inhibitor cocktail) for 3 hrs at 4°C with mixing. The resin was then washed with 1 ml of binding buffer four times and proteins eluted in 2X SDS loading buffer. 10% of the elution was loaded onto 15% SDS-PAGE and subjected to Coomassie staining. 90% of the elution was analyzed by western blotting with anti-FLAG antibody.

### In vitro SUMO binding assay with BRLF1

10 cm dishes of 293T were transfected with 8 μg each pCMV3FC or pCMV3FC-BRLF1 using 24 μl of PEI according to the manufacturer’s protocol. Cells were harvested 48 hours later and lysed in 50 mM Tris pH 8.0, 1 M NaCl, 0.1% sodium deoxycholate, 0.5% NP40, 2 mM EDTA, protease inhibitor cocktail (P8340; Sigma). Cells were lysed by sonication and lysates clarified by centrifugation. Protein concentrations were adjusted to 6.5 mg/ml and 600 μl of lysate was incubated with 5 μl equilibrated anti-FLAG M2 resin (Sigma A2220) for 2 hours. The resin was washed twice with 1 ml lysis buffer, twice with 1 ml BC100 (20mM Tris pH7.9, 100mM NaCl, 10% glycerol, 0.2mM EDTA, 0.2% TritonX100, protease inhibitor cocktail (Sigma P8849)) then blocked by 2 hr incubation in 1 ml BC100 containing 2% BSA. Bacterial lysates containing equivalent amounts of GST, GST-SUMO1 or GST-SUMO2 (generated as above except using BC100 lysis buffer) diluted to 100 μl in BC100/2%BSA were then added to the FLAG resin. The resin was washed five times with 1 ml BC100 containing 1% TritonX-100 and eluted in 2X SDS loading buffer. 20% of the elutions and 20% of the equivalent GST inputs were run anaylsed by SDS-PAGE and Coomassie staining to compare levels of input proteins. The remaining elutions (and 1% of the GST inputs) were analysed by Western blotting using anti-GST antibody and goat-anti rabbit secondary antibody (SAB3700878; 1:5000).

### *In vitro* SUMOylation of p53

Assays for *in vitro* SUMOylation of p53 were performed using the SUMOylation Assay Kit from Abcam (ab139470), as suggested by the manufacturer. 10 μl reactions included 50 ng of recombinant p53 as substrate (purified from *E*.*coli* as indicated above) and varying amounts (~100, 200 and 400 ng) of recombinant His6-SM-3FLAG, His6-UL54-3FLAG or His6-UL69-3FLAG (partially purified from *E*.*coli* as described above). Reactions with no viral proteins or with viral proteins but no SAE were included as negative controls. Reactions were incubated 1.5 hrs at 37°C and stopped by the addition of 2X SDS loading buffer. The presence of viral proteins and the SUMOylation of p53 were evaluated by Western blotting with FLAG, p53, SUMO1 and SUMO2 antibodies.

### SUMOylation of p53 in human cells

293T cells were co-transfected with plasmids expressing His6-SUMO1 or His6-SUMO2 and pCMV3FC expressing FLAG-tagged SM, UL54 or UL69 or empty pCMV3FC or positive control LMP1 and ICP0 plasmids as describe above for the SUMO screen. 36 hrs post transfection, 10% of the cells were lysed in 2X SDS loading buffer (input fraction) and 90% of the cells were lysed in lysis buffer G followed by purification of His-tagged SUMO conjugates on metal chelating resin as described above. Inputs and purified fractions were analyzed by western blotting with p53 and FLAG antibodies.

### SM expression and silencing in AGS-EBV cells

AGS-EBV cells in 6-well dishes were transfected with 100 pmoles of SM-specific siRNA (5’-GCUGCACCGAUGAAAGUUATT-3’) or AllStars negative-control siRNA (Qiagen) using 2 μl of Lipofectamine 2000 (Thermo Fisher Scientific). Two additional rounds of silencing were performed after 24 and 48 hours. Twenty-four hours later, cells were treated with 3 mM sodium butyrate (NaB) and 20 ng/ml 12-*O*-tetradecanoylphorbol-13-acetate (TPA) to induce the lytic cycle and harvested at 0, 8,16, 24 and 48 hours post- treatment for reverse-transcriptase quantitative PCR (qRT-PCR) of SM transcripts and Western blot analysis. For qRT-PCR, total RNA was extracted using TRIzol (Invitrogen) according to the manufacturer’s instructions. One microgram of total RNA was treated with 0.5 units of DNase I (New England BioLabs) for 15 min and reverse transcribed in a 20 μl reaction mixture using the SuperScript IV reverse transcriptase (Invitrogen) with random hexamer primers according to the manufacturer’s instructions. qRT- PCR was performed with 1 μl of 1:10 dilution of the cDNA using Luna Universal qPCR Master Mix (New England BioLabs) with a total reaction volume of 10 μl in a Bio-Rad CFX384 Real-Time System (Bio-Rad). Primers used were: SM forward 5’-CCTGCTTCCTTCCTAACACG-3’, SM reverse 5’-CGTGCCAGGGTTGTAATTCT-3’, β-actin forward 5′-GGACTTCGAGCAAGAGATGG-3′ and β-actin reverse 5′- AGCACTGTGTTGGCGTACAG-3′. The relative mRNA expression level was derived from 2-ΔΔCT by use of the comparative threshold cycle (CT) method. The amount of mRNA in each sample was normalized to the amount of actin mRNA. For Western blot analyses, Cells were lysed in 9 M urea-10 mM Tris (pH 6.8) followed by sonication and clarification by centrifugation. Twenty micrograms of clarified lysates were analysed by 8% SDS-PAGE and Western blotting using antibodies against SUMO1 (rabbit, 1:1000 dilution, sc-9060, Santa Cruz) or β-actin (mouse, 1:10,000 dilution, sc-47778, Santa Cruz) and secondary antibodies goat-anti-rabbit (1:5000 dilution, SAB3700878-1, Sigma) or goat anti-mouse (1:5000 dilution, sc-2005, Santa Cruz).

## Supporting information

S1 Fig Screens of EBV proteins for global effects on cellular SUMO1 and SUMO2 modifications. Western blots from Fig 1A and 1B but including blots for LMP1 (HA antibody; Cell Signalling #3724) and ICP0.
(TIF)

S2 Fig SM and BMRF1 are SUMO2-modified. 293T cells in 6 well dishes were co-transfected with plasmids (0.5 μg each) expressing FLAG-tagged SM or BMRF1 and His6-SUMO1, His6-SUMO2 or empty vector. Cells were harvested 36 hrs post transfection. 10% of the cells were lysed in 2X SDS loading buffer (60 mM Tris.HCl pH 6.8, 1% SDS, 100 mM DTT, 5% glycerol) to provide the input sample. 90% of the cells were resuspended in 0.5 ml lysis buffer G (6 M guanidine hydrochloride, 10 mM Tris, 100 mM sodium phosphate, pH 8.0) and subjected to purification of His-tagged SUMO conjugates as described in Materials and Methods. Inputs and purified fractions were analyzed by Western blotting with FLAG antibody. Arrowheads indicate non-modified SM or BMRF2. Stars indicate SUMO conjugated SM or BMRF1.
(TIF)

S3 Fig SM, UL54, UL69 and BRLF1 do not affect SUMO transcripts. 293T cells in 6 cm dishes were transfected with 2.5 μg of pCMV expressing SM, UL54, UL69, BRLF1 or empty pCMV (EV). 36 hrs later, total RNA was isolated from cells using the Trizol regent (Life Technologies). 1 μg of total RNA was reverse transcribed in a 25 μl reaction using SuperScript IV reverse transcriptase (Life Technologies) and random hexamer primers as suggested by the manufacturer. Quantitative real time PCR was performed according to the manufacturer’s recommendation using 1 μl of a 1:10 dilution the cDNA and Luna Universal qPCR mix (New England Biolabs) with a total reaction volume of 10 μl in a Bio-Rad CFX384 Real-Time System (Bio-Rad). Primers used to quantify mRNA levels were: SUMO1 forward 5′- GGGAAGGGAGAAGGATTTGTAA-3′, SUMO1 reverse 5′- GTCCTCAGTTGAAGGTTTTGC-3′, SUMO2 forward 5′-GCAGACGGGAGGTGTCTACT-3′, SUMO2 reverse 5′-AGTCAGGATGTGGTGGAACC-3′, Ubc9 forward 5’-ATTATCCATCTTCGCCACCA-3’, Ubc9 reverse 5’-TCTTGCCAAACCAATCCCT-3’, β-actin forward 5′-GGACTTCGAGCAAGAGATGG-3′ and β-actin reverse 5′-AGCACTGTGTTGGCGTACAG-3′. The relative mRNA expression level was derived from 2−ΔΔCT by use of the comparative threshold cycle (CT) method. The amount of mRNA in each sample was normalized to the amount of actin mRNA. The average values (with standard deviation) from two independent experiments are shown for SUMO1 (A), SUMO2 (B) and Ubc9 (C). A positive control for induction of these transcripts is also shown, generated by treatment of cells with the empty plasmid (EV) with sodium butyrate and TPA (Bu/TPA).
(TIF)
