# Supplementary figures and images for: A genome-wide screen of Epstein-Barr virus proteins that modulate host SUMOylation identifies a SUMO E3 ligase conserved in herpesviruses
Source: PLoS Pathog. 2018 Jul 6;14(7):e1007176. doi: 10.1371/journal.ppat.1007176 (PMC6051671; doi:10.1371/journal.ppat.1007176)

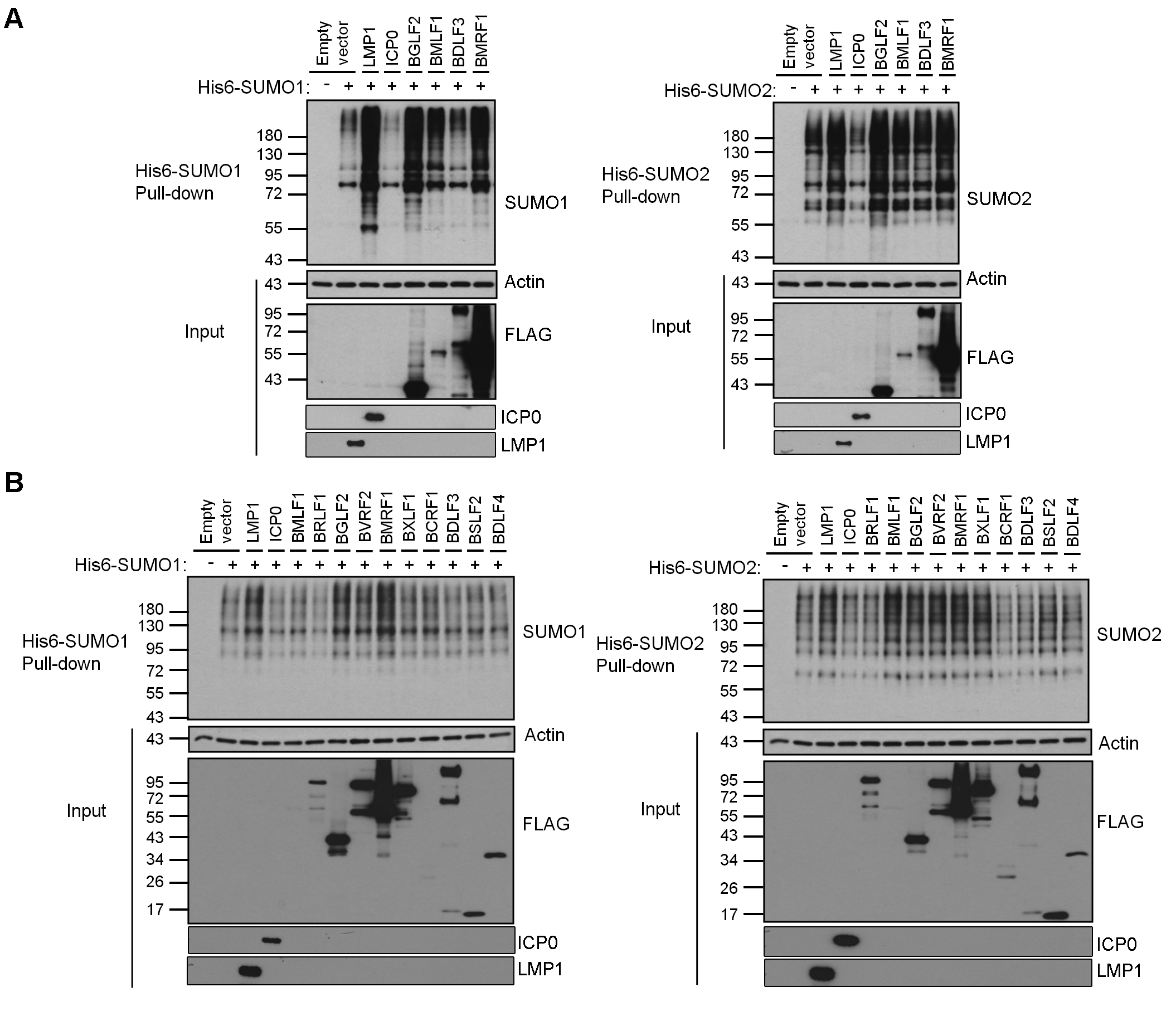

Supplement: S1 Fig — Western blots from Fig 1A and 1B but including blots for LMP1 (HA antibody; Cell Signalling #3724) and ICP0. (TIF) [file ppat.1007176.s001.tif]

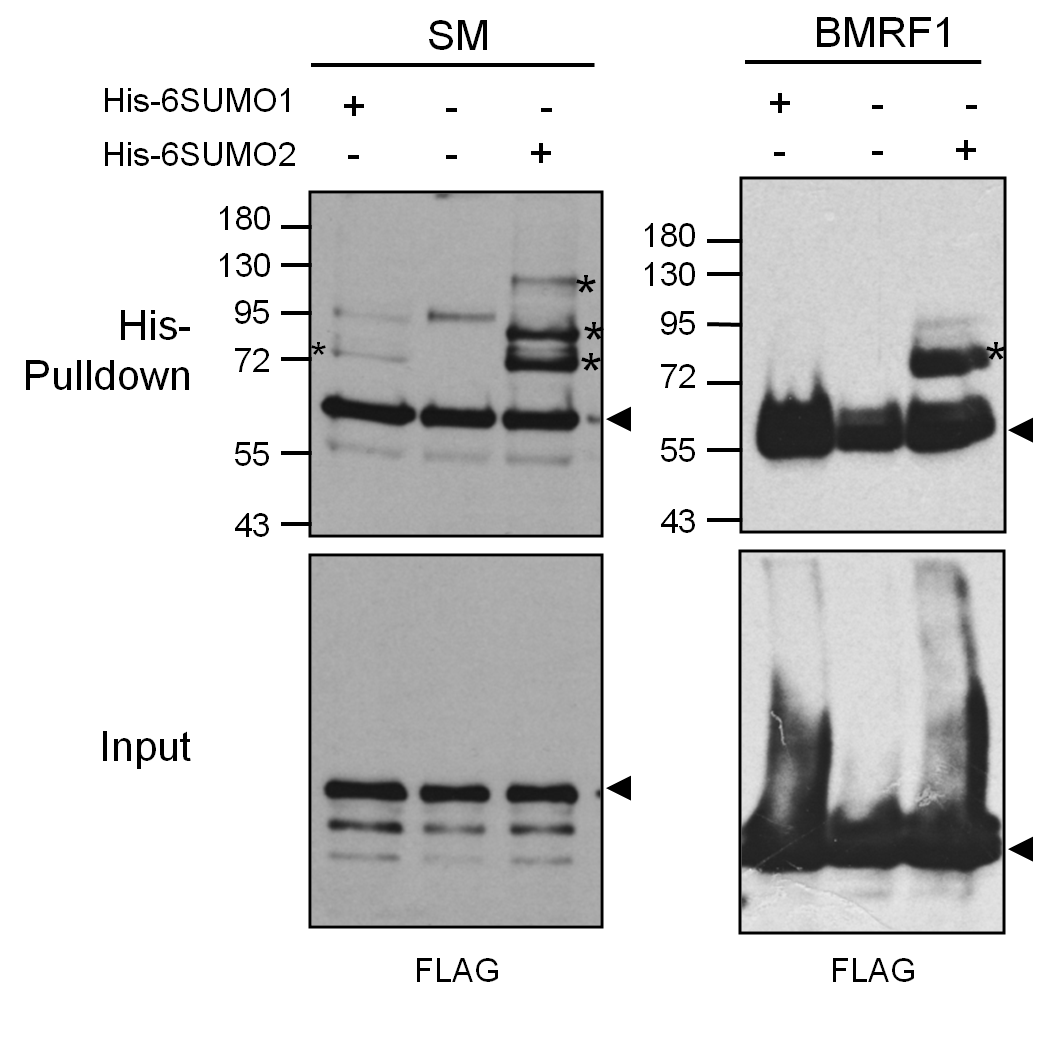

Supplement: S2 Fig — 293T cells in 6 well dishes were co-transfected with plasmids (0.5 μg each) expressing FLAG-tagged SM or BMRF1 and His6-SUMO1, His6-SUMO2 or empty vector. Cells were harvested 36 hrs post transfection. 10% of the cells were lysed in 2X SDS loading buffer (60 mM Tris.HCl pH 6.8, 1% SDS, 100 mM DTT, 5% glycerol) to provide the input sample. 90% of the cells were resuspended in 0.5 ml lysis buffer G (6 M guanidine hydrochloride, 10 mM Tris, 100 mM sodium phosphate, pH 8.0) and subjected to purification of His-tagged SUMO conjugates as described in Materials and Methods. Inputs and purified fractions were analyzed by Western blotting with FLAG antibody. Arrowheads indicate non-modified SM or BMRF2. Stars indicate SUMO conjugated SM or BMRF1. (TIF) [file ppat.1007176.s002.tif]

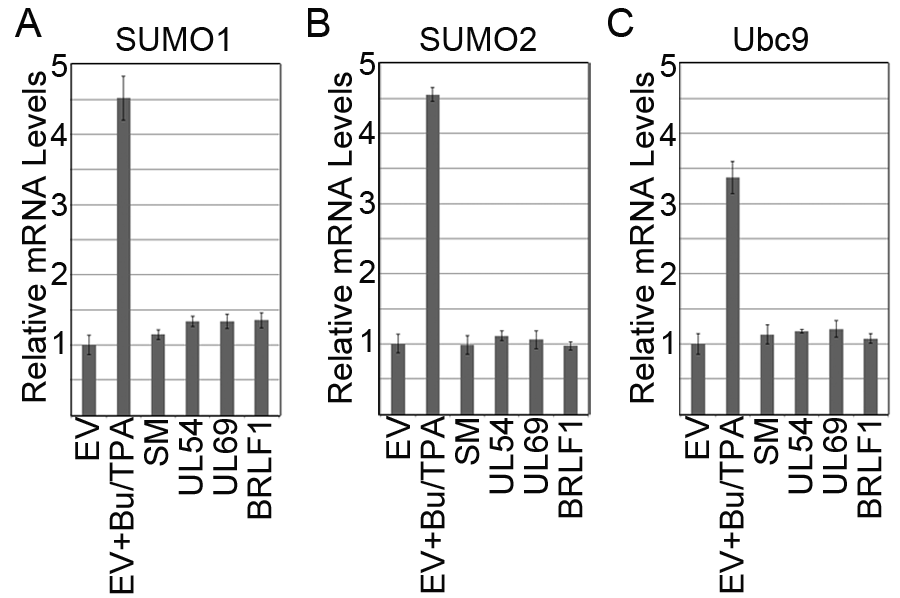

Supplement: S3 Fig — 293T cells in 6 cm dishes were transfected with 2.5 μg of pCMV expressing SM, UL54, UL69, BRLF1 or empty pCMV (EV). 36 hrs later, total RNA was isolated from cells using the Trizol regent (Life Technologies). 1 μg of total RNA was reverse transcribed in a 25 μl reaction using SuperScript IV reverse transcriptase (Life Technologies) and random hexamer primers as suggested by the manufacturer. Quantitative real time PCR was performed according to the manufacturer’s recommendation using 1 μl of a 1:10 dilution the cDNA and Luna Universal qPCR mix (New England Biolabs) with a total reaction volume of 10 μl in a Bio-Rad CFX384 Real-Time System (Bio-Rad). Primers used to quantify mRNA levels were: SUMO1 forward 5′- GGGAAGGGAGAAGGATTTGTAA-3′, SUMO1 reverse 5′- GTCCTCAGTTGAAGGTTTTGC-3′, SUMO2 forward 5′-GCAGACGGGAGGTGTCTACT-3′, SUMO2 reverse 5′-AGTCAGGATGTGGTGGAACC-3′, Ubc9 forward 5’-ATTATCCATCTTCGCCACCA-3’, Ubc9 reverse 5’-TCTTGCCAAACCAATCCCT-3’, β-actin forward 5′-GGACTTCGAGCAAGAGATGG-3′ and β-actin reverse 5′-AGCACTGTGTTGGCGTACAG-3′. The relative mRNA expression level was derived from 2−ΔΔCT by use of the comparative threshold cycle (CT) method. The amount of mRNA in each sample was normalized to the amount of actin mRNA. The average values (with standard deviation) from two independent experiments are shown for SUMO1 (A), SUMO2 (B) and Ubc9 (C). A positive control for induction of these transcripts is also shown, generated by treatment of cells with the empty plasmid (EV) with sodium butyrate and TPA (Bu/TPA). (TIF) [file ppat.1007176.s003.tif]
